# A Holistic Approach to Identifying a Positron Emission Tomography (PET) Tracer Candidate for In Vivo Imaging of Purinergic P2X7 Receptor in Neuroinflammation

**DOI:** 10.1021/acsptsci.5c00820

**Published:** 2026-03-29

**Authors:** Imane Ghafir El Idrissi, Andrea Spinaci, Daniele Vitone, Francesca Intranuovo, Mauro Niso, Leonardo Brunetti, Beatrice Francucci, Burcu A. Pazarlar, Kristin H. Magnusdottir, Eleonora Paradies, Carlo Marya Thomas Marobbio, Ludovica Ricci, Marianna Grignolo, Rosa Maria Iacobazzi, Gabriella Marucci, Diego Dal Ben, Catia Lambertucci, Rosaria Volpini, Nunzio Denora, Elena Adinolfi, Jens D. Mikkelsen, Michela Buccioni, Enza Lacivita, Marcello Leopoldo

**Affiliations:** a Dipartimento di Farmacia-Scienze del Farmaco, via Orabona, 4 Bari 70125, Italy; b Scuola di Scienze del Farmaco e dei Prodotti della Salute, Università degli Studi di Camerino, via Madonna delle Carceri, s.n.c., Camerino 62032, Italy; c Neurobiology Research Unit, University Hospital Rigshospitalet, Copenhagen 2100, Denmark; d Institute of Neuroscience, University of Copenhagen, Copenhagen 2200, Denmark; e CNR Institute of Biomembranes, Bioenergetics and Molecular Biotechnologies (IBIOM), Bari 70125, Italy; f Dipartimento di Bioscienze, Biotecnologie e Ambiente, via Orabona, 4,Bari 70125, Italy; g Dipartimento di Scienze Mediche, Sezione di Medicina Sperimentale, 165478 Università degli Studi di Ferrara, via Luigi Borsari, 46, Ferrara 44121, Italy

**Keywords:** neuroinflammation, positron emission tomography, purinergic P2X7 receptor, binding affinity, human, autoradiography

## Abstract

The central role of neuroinflammation in the pathogenesis of neurodegenerative diseases and brain disorders has spurred the development of Positron Emission Tomography (PET) radiotracers to investigate neuroimmune mechanisms noninvasively in vivo. Because it is expressed in glia, the purinergic P2X7 receptor (P2X7R) is a validated target for in vivo imaging of neuroinflammation, an alternative to the 18 kDa translocator protein, which is currently the standard target for neuroinflammation in clinical practice. However, clinically validated P2X7R PET radiotracers remain needed. The present study aimed to identify a novel molecular scaffold for developing an effective P2X7R PET radiotracer, starting from three chemotypes with antagonist activity in the nanomolar range at human P2X7R, to exploit structural diversity and meet the multidimensional key attributes that a CNS PET radiotracer must have. Thus, we evaluated the selected chemotypes across a range of properties, including radioligand binding affinity at the human P2X7 receptor, off-target selectivity, in vitro metabolic stability, and nonspecific binding to brain tissue. Our study pointed to compound **2** (2-chloro-3-methoxy-*N*-[2-morpholino-2-[6-(trifluoromethyl)­pyridin-3-yl]­ethylbenzamide) as a promising molecular scaffold to deliver an effective PET tracer because of its nanomolar affinity for human cloned P2X7R, broad off-target selectivity, high in vitro metabolic stability, passive permeability across two model membrane monolayers, limited interaction with blood-brain barrier efflux transporters, and brain free fraction predictive of low in vivo nonspecific binding. To ensure robust translatability, we also evaluated the binding affinity of compound **2** in human meningiomas by autoradiography and found that the compound binds to native P2X7R with high affinity (IC_50_ = 72 nM).

## Graphical abstract

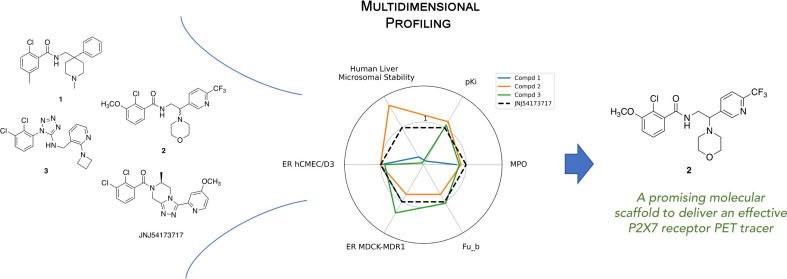

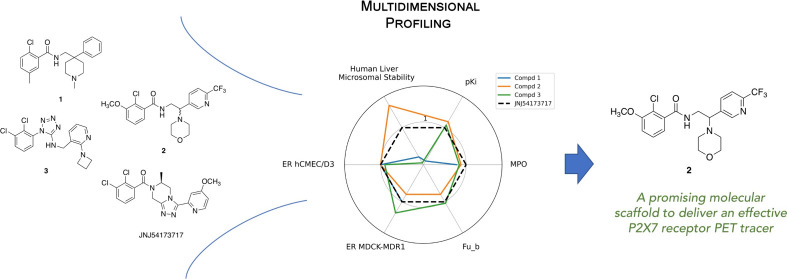

The term neuroinflammation refers to the complex immune response that occurs within the brain in reaction to different insults, such as infection, protein aggregation, trauma, or toxic injury.^1^ Microglia and astrocytes, the immune-competent cells of the brain, are key regulators of neuroinflammation and play a crucial role in maintaining neuronal function under homeostatic conditions.^2^ In pathological conditions, following chronic insults, glial cells become overactivated and begin to release neurotoxins, which activate and sustain a vicious cycle that culminates in neuronal death.^3^ It is not surprising that, over the last few decades, the interest in neuroinflammation has grown exponentially, and, consequently, the need to study neuroinflammation in vivo in human patients has emerged.^4,5^ Immune cell activation states are context-dependent; thus, having the possibility of longitudinally visualizing the neuroinflammatory processes in the same subjects can provide insights into the dynamic roles of glial cell activation in the pathological condition,^4^ also because neuroinflammation can occur a long time after the initial insult. Positron Emission Tomography (PET) offers significant potential in this context because it is a noninvasive imaging technique that can visualize and quantify biochemical processes in real time.^6,7^

PET imaging of neuroinflammation has, to date, used the 18 kDa translocator protein (TSPO) as a target, a protein located on the outer mitochondrial membrane. TSPO is involved in regulating the activation of both microglia and astrocytes and TSPO-based PET imaging has provided novel insights into the neurobiological underpinnings of neuroinflammation in CNS disorders.^8–10^ However, the lack of cellular specificity of TSPO, which is also expressed in platelets and endothelial cells and the inability to distinguish among the different activation phenotypes of microglial cells limit the interpretation of the results, blurring the distinction between pathological neuroinflammation and compensatory glial response.^11^ Therefore, several targets expressed in glia have been proposed as relevant biomarkers to quantify different aspects of neuroinflammation specifically.^5,12^ Among these is the purinergic P2X7 receptor (P2X7R), an ATP-gated ion channel belonging to the purinergic P2X receptor family, that is highly expressed in microglia, oligodendrocytes and astrocytes.^13^ P2X7R expression is specifically increased in microglia in pro-inflammatory environments and, thus, it has been proposed as a target for visualizing pro-inflammatory processes.^14–16^

Over the years, several P2X7R ligands have been radiolabeled and evaluated as potential PET radioligands (Figure 1). Most of them have demonstrated poor metabolic stability or low brain uptake in preclinical models or in humans, including [^11^C]-A740003,^17^ [^18^F]-EFB,^18^ and [^11^C]-GSK1482160.^19^ Instead, [^11^C]-SMW139, [^11^C]-JNJ54173717, and [^18^F]-JNJ64413739 have shown significant brain uptake in humans. [^11^C]-SMW139 demonstrated increased in vivo binding potential in patients with relapsing remitting multiple sclerosis compared with age-matched healthy individuals.^20^ In another study, [^11^C]-SMW139 showed increased P2X7R binding in the putamen of patients with Parkinson’s Disease (PD) compared with healthy controls, suggesting an increase in proinflammatory processes in PD.^21^ However, the short half-life of carbon-11 (20 min) limits the widespread use of [^11^C]-SMW139. Differently, [^11^C]-JNJ54173717 was not able to discriminate between PD patients and healthy controls because of high between-subject variability, most likely related to P2X7R polymorphisms.^22,23^ [^18^F]-JNJ64413739 displayed promising biodistribution in healthy individuals, but no data in pathological conditions have been reported yet. Therefore, to date, no clinically validated P2X7R PET radiotracer has been identified.^24^

**Figure 1:**
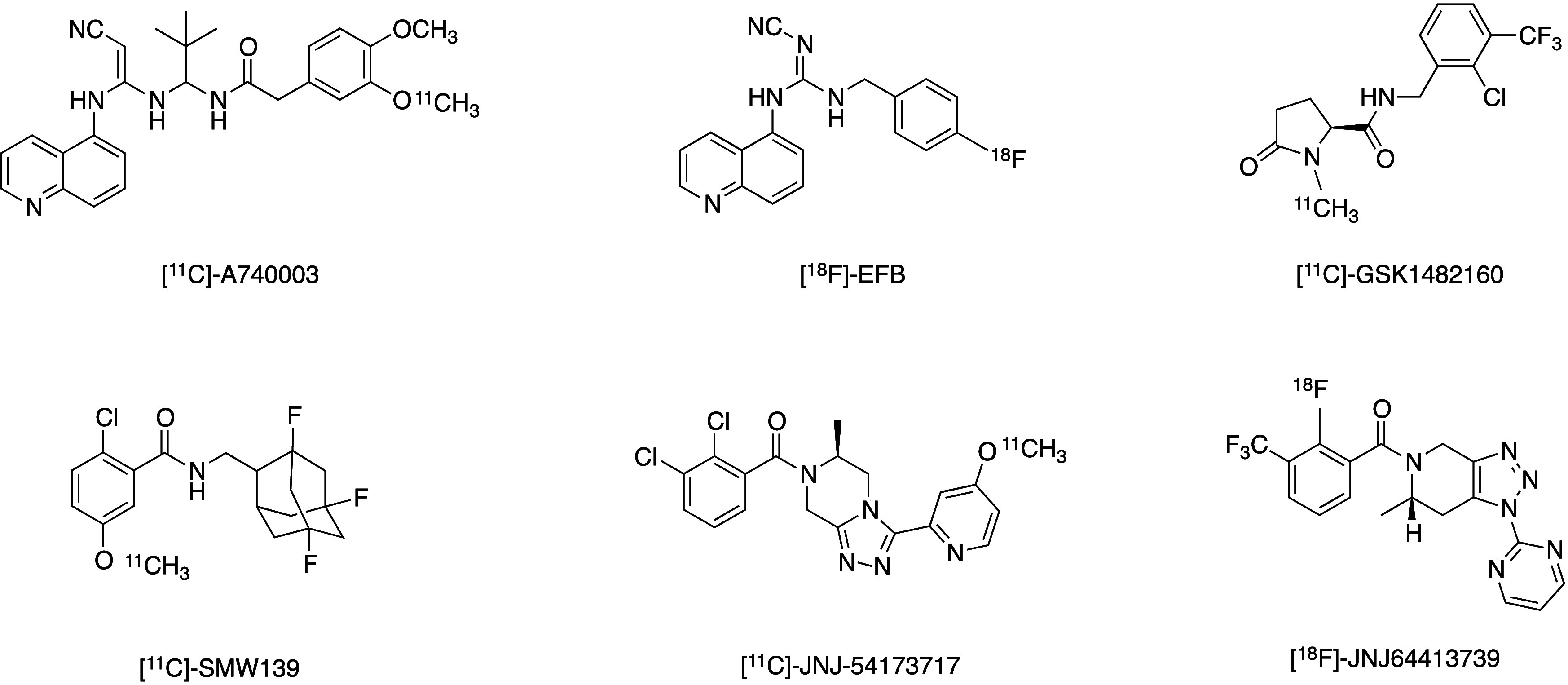
P2X7R-targeting PET radioligands.

CNS PET imaging requires high quality ligands; that is, they must meet specific criteria to achieve precise targeting of biological targets and to generate clear, high-contrast images. The ligand should *i)* contain a structural moiety amenable for positron emitting isotope incorporation; *ii)* have high affinity (*B*_max_/K_d_ > 10) and selectivity (typically higher than 30-fold) for the target; *iii)* be brain penetrant and not form brain-permeable radioactive metabolites; *iv)* have low nonspecific binding to brain tissue to achieve a sufficient signal-to-noise ratio for quantification.^25^

In this study, we applied a holistic approach to identify a novel molecular scaffold for developing an effective P2X7R PET radiotracer through a multidimensional profiling of the key attributes of a CNS PET tracer. We selected three different chemotypes (Figure 2) with nanomolar activity at the human P2X7R and evaluated their binding affinity at human and rat P2X7R, selectivity, metabolic stability, and nonspecific binding to brain tissue. We also evaluated the binding affinity of the selected compounds in human meningioma tissues by autoradiography as a measure of their ability to bind native human P2X7R.

**Figure 2:**
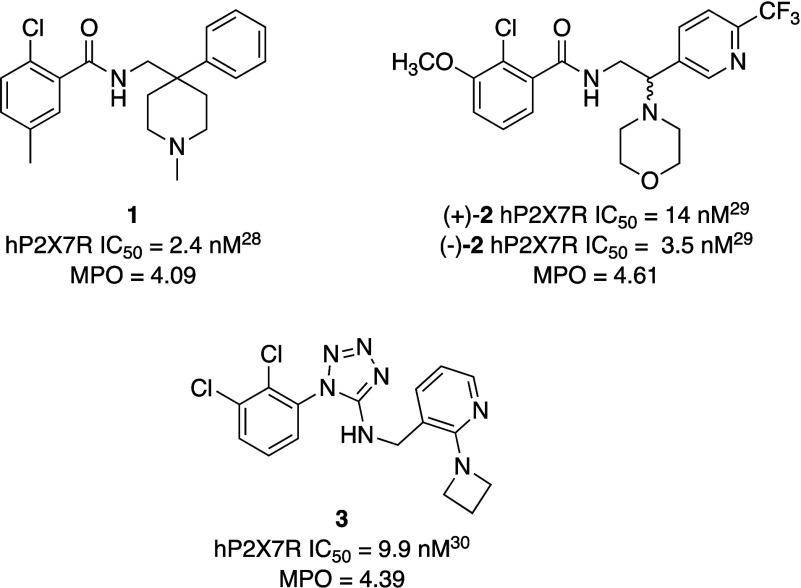
Structural formulas of the selected P2X7R antagonists and their functional activity at hP2X7R.

## Results and Discussion

To identify a molecular scaffold that could deliver an effective PET radioligand, we browsed the literature, including patent applications, looking for compounds featuring an IC_50_ lower than 10 nM for human P2X7R activity, tractable chemistry, and favorable physicochemical properties compatible with a PET radiotracer. For the analysis of physicochemical properties, we used the MultiParameter Optimization (MPO) tool. MPO enables tracking of six physicochemical properties (cLogP, cLogD, molecular weight, topological polar surface area, number of hydrogen-bond donors, and p*K*_a_) commonly considered in drug development.^26^ As it has been reported that the majority (79%) of effective PET tracers has a MPO value >3,^25^ we selected compounds residing in this range.

The analysis of the literature revealed that the ortho-substituted arylamide is a privileged scaffold for P2X7R binding, where an ortho-chloro substituent and a lipophilic moiety linked to amide function are preferred to obtain nanomolar affinity.^27^ Consistently, [^11^C]-SMW319, [^11^C]-JNJ54173717, and [^18^F]-JNJ64413739 present such chemical features.

Our literature search returned three different chemotypes, i.e., compounds **1**, (+)- and (−)-**2**, and **3** (Figure 2), that had IC_50_ lower than 10 nM at human P2X7R and MPO values higher than 3 (Figure 2). Compounds **1** and **2** share the 2-chlorobenzamide moiety but differ in the moieties linked to the amide function.^28,29^ Polar functionalities, such as morpholino or *N*-methylpiperidine rings, are incorporated into these moieties, thereby allowing modulation of the lipophilicity of the molecule. Instead, compound **3** features a bioisosteric replacement of the benzamide moiety, namely the 1,2,4-tetrazole ring.^30^ In compound **3**, the polar functionalities (azetidine and pyridine rings) are present on the moiety linked to the 1,2,4-tetrazole ring. Given that the two enantiomers of compound **2** exhibited comparable functional activity at hP2X7R,^29^ we decided, at this stage, to study the racemic mixture due to its synthetic accessibility. Thus, we assessed the functional activity and binding affinities of compounds **1–3**, and evaluated their pharmacokinetics to assess their developability potential as PET radiotracers. In parallel, we tested JNJ54173717 for comparative purpose because of its excellent brain uptake in humans.

### Chemistry

Compounds **1**, **2**, and **3**, and JNJ-54173717 were prepared as depicted in Schemes 1 and 2, following the procedure reported in the literature with minor modifications.^28–31^ The synthesis of compound **1** started from the commercially available phenylacetonitrile **4**, which was condensed with the N,N-bis­(2-chloroethyl)-*N*-methylamine **5** to obtain the nitrile **6**. Hydrogenolysis of nitrile **6** in the presence of Raney nickel as catalyst gave amine **7**, which was condensed with 2-chloro-5-methylbenzoic (**8**) acid to provide **1** (Scheme 1). For the preparation of compound **2**, aldehyde **9** reacted with trimethylsilylcyanide (TMSCN) and morpholine to give nitrile **10**, which was hydrogenated in the presence of Raney nickel as catalyst to give amine **11**. The target compound **2** was prepared by condensing amine **11** with 2-chloro-3-methoxybenzoic acid (**13**), which was prepared by oxidazing aldehyde **12** (Scheme 1). The synthesis of JNJ54173717 started from the commercially available (*S*)-*t*-butyl 2-methyl-5-oxopiperazine-1-carboxylate (**14**), which was activated with (CH_3_)_3_OBF_4_ and then condensed with the 4-methoxypyridin-2-ylhydrazide to yield compound **15**. The latter compound was subjected to BOC deprotection to provide amine **16**, which was condensed with the 2,3-dichlorobenzoyl chloride (**17**) to afford JNJ54173717. The synthesis of compound **3** required the preparation of bromo derivative **20** and amine **23** (Scheme 2). 2,3-Dichlorophenylhydrazine (**18**) reacted with formamide at 150 °C to provide the triazole **19**, which was brominated under radical conditions to afford the bromo derivative **20**. Amine **23** was prepared starting from 3-cyano-2-fluoropyridine **21**, which, following aromatic nucleophilic substitution reaction by azetidine, gave nitrile **22**. The latter was hydrogenated in the presence of Raney nickel as a catalyst to afford amine **23**, which, following solid fusion with bromo derivative **20**, gave compound **3**. The experimental procedures and the spectroscopic data for the intermediates are reported in the Supporting Information.

**Scheme 1:**
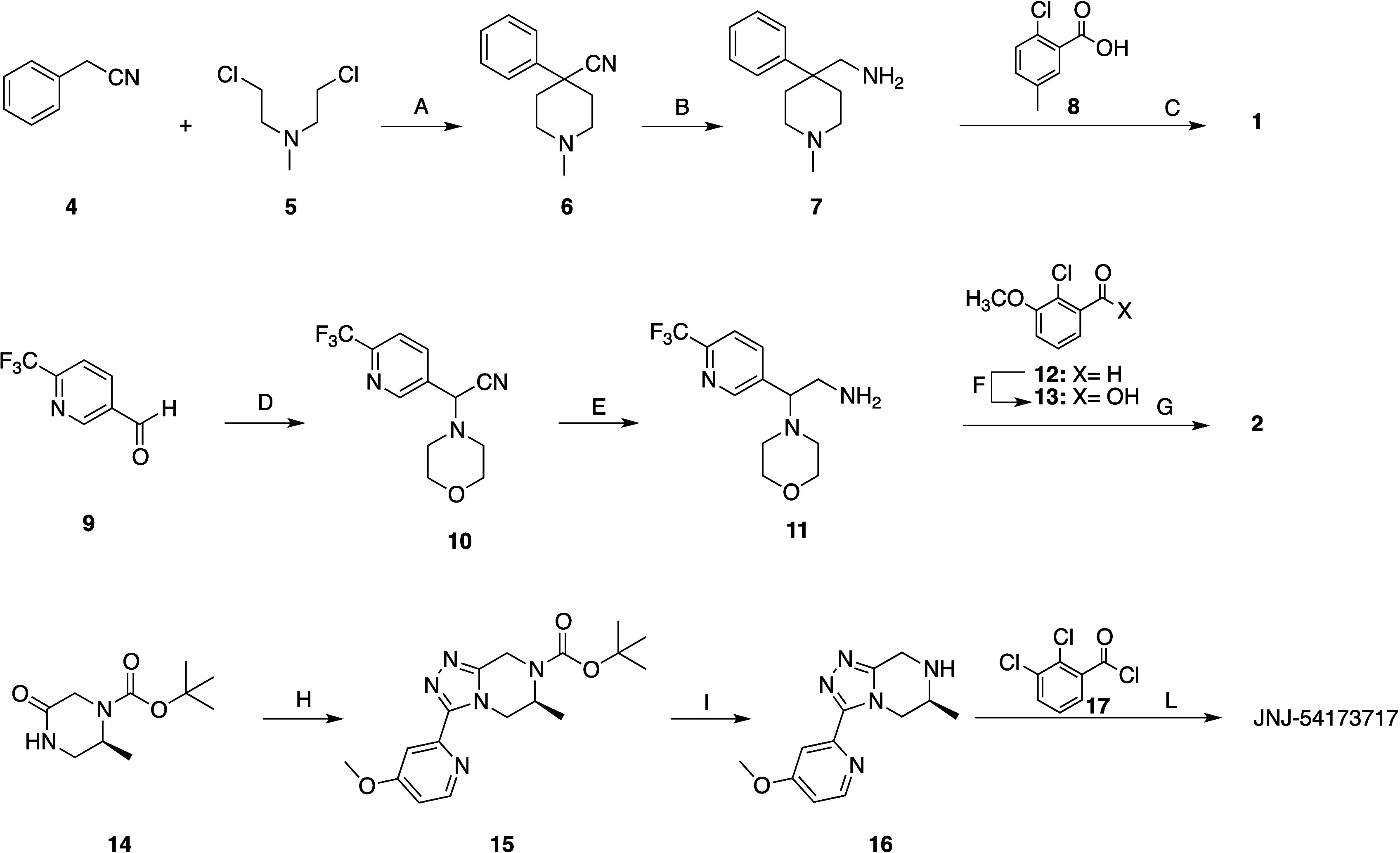
Synthesis of Compounds **1** and **2** and JNJ-54173717^a^

a Reagents and conditions: (A) NaH, anhydrous DMF, 70 °C, overnight, 75% yield; (B) H_2_, Nickel Raney, 5 atm, 40 °C, 22 h, quantitative yield; (C) PyBOP, *N*-methylmorpholine, anhydrous DMF, room temperature, 75% yield; (D) Morpholine, TMSCN, t.a., 12 h, HOAc, 78% yield; (E) H_2_, 30 bar, Ni-Raney, EtOH, 50 °C, 0.3 mL/min, 22% yield; (F) KMnO_4_, saturated sol. NaHCO_3_, 90 °C, 5 h, 81% yield; (G) EDC, HOBt, DIPEA, DMF, 25 °C, 12 h, 52% yield; (H) (i) (CH_3_)_3_OBF_4_, anhydrous DCM, r.t., 6 h; (ii) 4-methoxypyridin-2-ylhydrazide, DCM, r.t.; overnight; (iii) saturated NaHCO_3_, 90 °C, 6 h; 90% yield; (I) CF_3_COOH, DCM, r.t.; 45 min, 92% yield; (L) Et_3_N, DCM, r.t., 2 h, 48%.

**Scheme 2:**
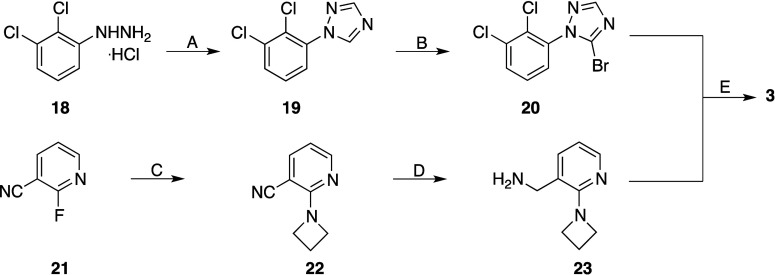
Synthesis of Compound **3** ^a^

a Reagents and conditions: (A) formamide, 150 °C, 16 h, 79% yield; (B) NBS, benzoyl peroxide, CCl_4_, 80 °C, 12 h, 60% yield; (C) azetidine, Et_3_N, 0–25 °C, THF, 96% yield; (D) H_2_, Raney nickel, 4 atm, NH_3_/MeOH 7N, 50 °C, 6 h, 87% yield; (E) solid fusion at 140 °C, 12 h, 20% yield.

### Binding Affinity at Human and Rat P2X7R

The majority of the known P2X7R antagonists are negative allosteric modulators that interact with an allosteric binding site formed between the neighboring subunits of the receptor and juxtaposed with the ATP binding pocket.^27^ The interaction of P2X7R ligands with this allosteric binding pocket has been confirmed by crystallographic and site-directed mutagenesis studies.^32^ Thus, P2X7R ligands are routinely characterized in functional assays, while the affinity of the molecule for the receptor is very seldom reported. As discussed in the Introduction, a PET radiotracer should have a high affinity for the target, and the required affinity (*K*_d_) depends on binding site density (*B*_max_). Generally, a *B*_max_/*K*_d_ ratio higher than 10 is needed for a successful PET radiotracer. Therefore, the candidate compound should have at least nanomolar affinity for the target receptor.

Based on these considerations, we assessed the binding affinity of the selected compounds **1–3** and the reference JNJ-54173717 at the human cloned P2X7R (hP2X7R) using a radioligand binding assay (Table 1). We selected [^3^H]-JNJ64415739 as a radioligand because we previously characterized its binding properties at the P2X7R in human brain tissues.^33^ Thus, we could easily compare the binding affinity data from the cloned receptor with those from human tissue (see below). We found that derivatives **2** and **3**, and JNJ-54173717 exhibited *K*_i_ values consistent with the published IC_50_ values.^29–31^ Compound **2** displayed subnanomolar affinity for hP2X7R (*K*_i_ = 0.75 nM), the highest among the studied compounds. Additionally, compound **3** and JNJ64415739 showed high hP2X7R affinity (*K*_i_ = 3.20 nM and *K*_i_ = 17 nM, respectively), although their binding affinity was lower than that of compound **2**. Instead, we were unable to assess specific binding for compound **1** up to 30 μM, although an IC_50_ value of 2.4 nM was reported in the patent application WO2014057078A1.^28^ Next, we assessed the functional activity of the three selected compounds in HEK-293 cells stably transfected with human P2X7R (Table 1, Figure S2 of the Supporting Information).^34^ We found that compounds **2** and **3** blocked Ca^2+^ mobilization induced by BzATP at nanomolar concentration, whereas compound **1** was inactive (IC_50_ > 5000 nM), consistent with the radioligand binding assay. It is, therefore, conceivable that the IC_50_ value reported in the patent application was incorrect. Of note, differently from binding studies, in our functional assay compound **3**, with an IC_50_ of 0.79 nM, was about fifty-fold more potent than compound **2** (IC_50_ = 33 nM).

**Table 1:** Binding Affinity and Functional Activity at Human and Rat P2X7R

compound	MPO	hP2X7R affinity	rP2X7R affinity	hP2X7R functional activity
		*K*_i_, nM	*K*_i_, nM	IC_50_, nM
**1**	4.09	>30,000 nM	>30,000	>5000
**(**±**)-2**	4.61	0.75 ± 0.04	438 ± 51	33 ± 2.2
**3**	4.39	3.20 ± 0.61	170 ± 7	0.79 ± 0.03
**JNJ54173717**	5.23	17 ± 1.3	13 ± 0.9	
**JNJ64413739** ^a^		12 ± 0.082		

a Kolb et al. reported a *K*i value of 15 nM for JNJ64413739.^39^

We also assessed the binding affinity of the selected compounds for rat P2X7R (rP2X7) (Table 1). Indeed, differences in function among human, rat, and mouse receptors have been demonstrated by several chemical series of P2X7R ligands, which may underlie a lack of species crossover. This is crucial because it can significantly impede interspecies translatability. We found that compounds **2** and **3**, unlike JNJ54173717, showed lower *K*_i_ values at the rP2X7R than at the human receptor. In particular, compound **2**, which had subnanomolar affinity at hP2X7R, showed *K*_i_ in the submicromolar range at rP2X7R. Compound **1** was not able to bind rP2X7R. The molecular bases of interspecies differences remain poorly understood. While the present manuscript was in preparation, Guo et al. proposed that the occupancy of the inner and more lipophilic part of the allosteric binding pocket, particularly the interaction with V312, may account for species-specific differences in P2X7R activity.^35^ On this basis, the binding interaction of compounds **2** and **3**, in comparison with JNJ-54173717, will be the object of future computational studies to verify this hypothesis.

P2X7R is highly polymorphic in humans and studies have linked single nucleotide polymorphisms (SNPs) of P2X7R to various disease states.^36,37^ The polymorphism rs3751143 (Glu496Ala), which is reported as a loss-of-function mutation, has been associated with Alzheimer’s Disease and PD.^38^ Based on the observation that JNJ54173717 failed to discriminate between PD patients and healthy volunteers, most likely because of the P2X7R polymorphism, we expressed the Glu496Ala mutant receptor in HEK-293 cells to evaluate the binding affinity of our compounds. We performed the assay using [^3^H]-JNJ64413739 as the radioligand and employed the experimental protocol used with the wild-type receptor. Although we tested a wide range of radioligand concentrations (from 1 to 400 nM), the saturation of the binding sites was not achieved. Saturation curves for human, rat and E496A mutant human P2X7Rs are presented in Figure S1 of the Supporting Information. These results suggest that the SNP not only influences the functional state of the receptor but also affects the binding properties of the allosteric binding site.

### In Vitro Pharmacokinetic Profiling of Compounds 1–3 and JNJ-54173717

In addition to high affinity and selectivity, an effective CNS PET radiotracer must have appropriate pharmacokinetics, be brain-penetrant, and exhibit minimal nonspecific binding.^40^ Therefore, we characterized the in vitro pharmacokinetic profile of the selected compounds by evaluating the metabolic stability in rat and human liver microsomes, the passive permeability in hCMEC/D3 and MDCK-MDR1 cells, and the unbound fraction in brain homogenate. We included JNJ54173717 as a reference for comparison.

We assessed metabolic stability in human and rat liver microsomes using a NADPH-regenerating system and evaluated the half-life (*t*_1/2_) and the intrinsic clearance (CL_int_). The compounds showed very different half-lives (Table 2). In fact, compound **3** was rapidly degraded in both rat and human microsomes (*t*_1/2_= 4.6 and 8.5 min, respectively). At the same time, compound **2** was stable to metabolic degradation in both species (*t*_1/2_= 165 and 266 min, respectively), which were longer than those observed for JNJ-54173717 (*t*_1/2_= 54 and 162 min, respectively).

**Table 2:** Metabolic Stability, Passive Permeability, and Unbound Brain Fraction of Compounds **1**–**3** and JNJ-54173717

	Microsomal stability				Passive permeability						Brain free fraction (f_u_)
	rat		human		hCMEC/D3			MDCK-MDR1			
comp.	*t*_1/2_ (min)	CL_int_ (μL/min/mg)	*t*_1/2_ (min)	CL_int_ (μL/min/mg)	P_app_ apical-to basolateral (10^–^^5^ cm/s)	P_app_ basolateral-to-apical (10^–^^5^ cm/s)	ER ratio	P_app_ apical-to basolateral (10^–^^5^ cm/s)	P_app_basolateral-to-apical (10^–^^5^ cm/s)	ER ratio	
**1**	18	38.5	35	19.8	3.5 ± 0.1	2.03 ± 0.03	0.6	2.6 ± 0.1	2.5 ± 0.1	0.9	not tested
**(**±**)-2**	165	4.2	266	2.6	4.5 ± 0.1	2.6 ± 0.1	0.6	4.2 ± 0.2	3.0 ± 0.7	0.7	20%
**3**	4.6	152	8.9	77.86	4.9 ± 0.1	2.7 ± 0.1	0.5	3.5 ± 0.1	4.2 ± 0.1	1.2	40%
**JNJ54173717**	54	12.8	162	4.27	4.7 ± 0.2	2.6 ± 0.1	0.6	4.1 ± 0.3	3.6 ± 0.1	0.9	35%

To predict the ability of the selected compounds to penetrate the blood-brain barrier (BBB) and to accumulate into the brain we assessed passive permeability and efflux ratio in the human brain microvascular endothelial cell line hCMEC/D3 cell monolayer, as a model of BBB, and in MDCK-MDR1 cell monolayer, the gold standard for assessing in vitro interaction with P-glycoprotein.

We assessed the apparent permeability (P_app_) across the cell monolayer from basolateral to apical (BA) and from apical to basolateral (AB) direction, and the efflux ratio (ER) between BA and AB fluxes. ER greater than 2 is predictive of undesirable interaction with the efflux transporters and, thus, limited brain permeation.^41^ We found that all compounds exhibited high permeation rates from the basolateral to the apical direction in both cell models, suggesting that they can diffuse passively across the cell membrane. Interestingly, the compounds also showed good permeation rates from the apical to basolateral direction, suggesting their permeability is not strongly influenced by the efflux transporters located on the apical membrane of the cell. Indeed, all the compounds showed ER values well below 2. The P_app_ values and ER ratio of compounds **1–3** are within the same range as those of JNJ-54173717 (Table 2). These results suggest that the compounds can cross the BBB in vivo and accumulate in the brain.

Low nonspecific binding (NSB) is another critical factor in the success of a brain-imaging PET radiotracer. NSB originates from the interaction of the molecule with nontarget proteins and phospholipids in cell membranes and correlates with the lipophilicity of the molecule.^42^ Among the different methodologies available to measure NSB in vitro, we decided to assess the brain free fraction (f_u,brain_) using equilibrium microdialysis with rat brain homogenate.^43^ It has been reported that a f_u,brain_ value greater than 15% is usually associated with low NSB in PET radiotracers.^25^ We found that both compounds **3** and **2** showed f_u,brain_ values higher than 15% (Table 2), thus suggesting that both compounds should have low NBS in vivo. The f_u,brain_ values correlate with the lipophilicity of the molecules. Interestingly, the f_u,brain_ values of compounds **2** and **3** were in the same range as that of JNJ54173717.

We graphically summarized the results of the multidimensional profiling using a radar plot (Figure 3) that compares MPO values, binding affinity, human liver microsomal stability, and efflux ratios in hCMEC/D3 and MDCK-MDR1 cells of compounds **1–3** to those of JNJ54173717. Considering the in vivo brain uptake and biodistribution of [^11^C]-JNJ54173717, we regarded this compound as our “reference tracer” for receptor affinity, selectivity, and pharmacokinetic properties (black dashed line in the graph). Properties better than those of JNJ54173717 are outside the black dashed line, while properties worse than those of JNJ54173717 are inside. The compounds differ mainly in receptor affinity and metabolic stability, two crucial properties for an effective PET tracer. Compound **2**’s profile more closely resembles that of JNJ54173717, with a marked improvement in metabolic stability. Interestingly, compound **2** has binding properties, passive permeability, and efflux systems interaction comparable to those of the PET radioligands [^11^C]-SMW139, [^11^C]-JNJ54173717, and [^18^F]-JNJ64413739,^20,22,24^ which showed good brain uptake in humans. In addition, compound **2** outperforms the above PET radioligands in terms of microsomal stability in rat and human liver microsomes, thus suggesting it as a promising structural scaffold for developing effective P2X7R PET radioligands.

**Figure 3:**
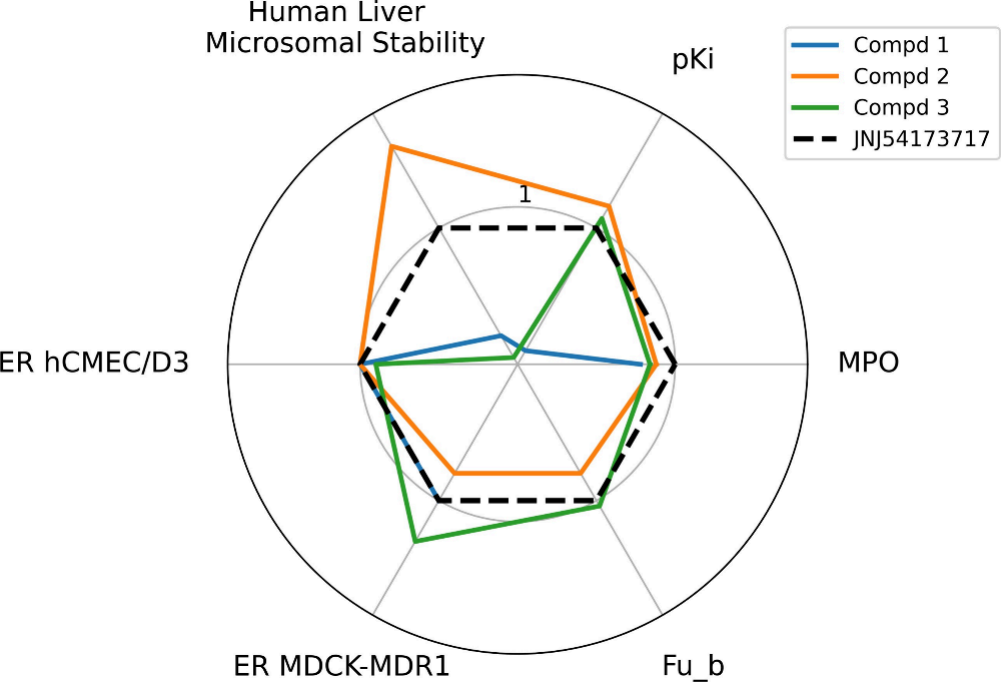
Radar plot comparing the affinity and pharmacokinetic profiles of compounds **1–3** with that of JNJ54173717. Since each property has a different measurement unit, to make the graph easier to read, we assigned a value of 1 to each property of JNJ54173717 and then calculated the number of folds by which each property of compounds **1–3** differs from that of JNJ54173717.

### Autoradiography in Human Brain Samples

The binding properties of compounds **1–3** and JNJ54173717 were studied in human samples using [^3^H]-JNJ64413739 as the radioligand. It is anticipated that cloned receptors expressed in cell cultures may not fully replicate the binding properties in native tissues. Given that the structure of the native receptor in post-mortem human tissues may change due to post-mortem degeneration and this could affect binding properties, we used fresh human tissues expressing P2X7R. In particular, we used selected meningiomas obtained from surgical resections. We assessed P2X7R expression (*B*_max_) in meningiomas specimens using saturation analysis, and the tissues with the highest *B*_max_ were used for autoradiography. Table 3 lists the IC_50_ values obtained for compounds **1–3** and JNJ-54173717 and the relative binding (%), normalized to binding with [^3^H]-JNJ64413739 alone are in Figure 4. We found that compounds **2**, **3**, and JNJ-54173717 completely displaced [^3^H]-JNJ64413739 from the binding site. At the same time, compound **1** did not achieve 50% displacement of the radioligand at 1 μM, the highest test concentration, thereby confirming that the compound is unable to interact with P2X7R also in human tissue. In addition, while compound **3** and JNJ54173717 exhibited IC_50_ values comparable to the *K*_i_ values observed in radioligand binding affinity assay, compound **2** exhibited an IC_50_ value (IC_50_ = 72.44 nM) considerably higher than the *K*_i_ value (*K*_i_= 0.75 nM). This discrepancy between cloned and native receptor affinities might be due to several factors, including differences in binding-site accessibility between membrane homogenates and tissue slices,^44^ as well as differences in incubation time and/or radioligand concentration, which can make potential differences in the binding kinetics of the test compounds more evident.

**Table 3:** Binding Properties of Compounds **1–3** and JNJ-54173717 in Human Meningioma Samples

comp.	displacement of [^3^H]-JNJ64413739	IC_50_ (nM)	95% Cl (nM)
**1**	no	>1000 nM	420 nM to ∞
**(±)-2**	yes	72.44	44.2 to 128.7
**3**	yes	9.99	4.9 to 28.5
**JNJ54173717**	yes	9.89	5.12 to 20.65

**Figure 4:**
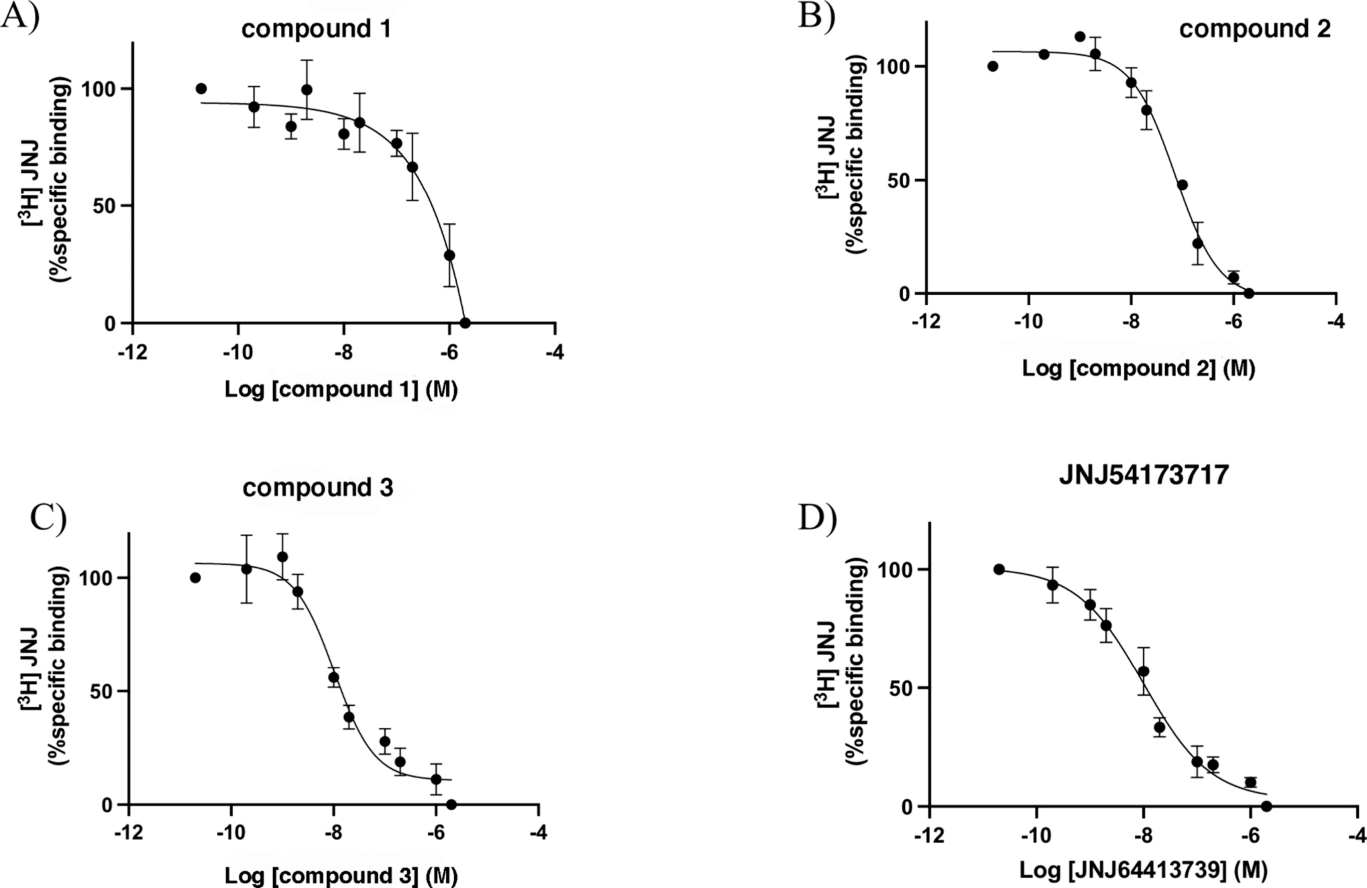
Displacement curves of compounds **1** (A), **2** (B), **3** (C) and JNJ54173717 (D) in human meningioma samples.

### Evaluation of Selectivity Profile

The biological characterization of the three selected compounds pointed to compound **2** as a scaffold for developing a P2X7R PET radiotracer because it has nanomolar affinity for human cloned P2X7R, is metabolically stable in liver microsomes, has good passive permeability and limited interaction with efflux transporters, brain free fraction higher than 15%, and can bind native P2X7R in human tissue. Based on these results, we evaluated the in vitro selectivity profile of 10 μM **2** against 46 targets (including CNS receptors and enzymes) and observed high selectivity for hP2X7R, with specific binding inhibition below 50% (see Table S1, Supporting Information).

## Conclusions

Neuroinflammation is a complex, dynamic process involving multiple cell types and characterized by well-defined spatiotemporal activation. In vivo PET imaging of neuroinflammation is a strategy that promises to clarify better the evolution of the inflammatory response in the brain and to investigate potential therapeutic targets. Developing an effective PET radiotracer is challenging because several stringent properties must be combined within a single molecule. In this study, we applied a holistic approach to identify a molecular scaffold that could deliver an effective PET radiotracer for in vivo imaging of P2X7R, a marker of activated microglia. To this end, we selected three chemotypes reported to bind hP2X7R and characterized them for binding affinity and selectivity at P2X7R, as well as for their in vitro pharmacokinetic profiles. Among the studied compounds, compound **2** emerged as a promising candidate exhibiting nanomolar affinity for human cloned and native P2X7R, broad selectivity, high metabolic stability, passive permeability, limited interaction with efflux transporters, and a brain free fraction predictive of low in vivo nonspecific binding. On the other hand, compound **2** has submicromolar affinity for rat P2X7R, which poses an important challenge for translation into preclinical studies. A potential strategy to overcome this limitation is the use of humanized transgenic P2X7R mouse models, which offer a valuable alternative for studying the pharmacology of human receptors in vivo.^45,46^ Nonetheless, we believe that additional important translation challenges warrant consideration. Several recent studies have highlighted species-specific differences in glial cells, which may have contributed to the failure to identify effective PET radiotracers for neuroinflammation imaging. Human microglia differ significantly from those of rodents in morphology, gene expression, and function.^12^ Thus, a paradigm shift in the development of PET tracers for neuroinflammation imaging is needed. In this regard, evaluation of affinity and selectivity profiles via autoradiography in human tissues (although not routinely accessible), in combination with dosimetry studies in rodents, to assess biodistribution and safety, could facilitate the identification of candidates for clinical PET studies. Compound **2** has a methoxy group that allows labeling with ^11^C. However, the studies on [^11^C]-SMW139 have demonstrated that the radionuclide’s short half-life limits the tracer’s application, as shorter-scan protocols are required, which may reduce the distribution volume of the tracer and make it more challenging to detect relatively small group differences.^21^ We believe that the molecular scaffold of compound **2** could deliver effective PET tracers, and future studies will focus on introducing ^18^F labeling into this scaffold. Ultimately, this study paves the way for the development of compounds based on the same chemical scaffold as potential brain-permeant therapeutics for a range of neurological conditions in which the P2X7R has been implicated, including Alzheimer’s disease^47^ and treatment-resistant brain tumors such as gliomas.^48,49^

## Experimental Section

### Chemistry

All reagents, solvents or silica were purchased from Merck (Milan, Italy), Acros (Fisher Scientific GmbH, Nidderau, Germany), Alfa Aesar (Thermo Fisher, Kandel), Carlo Erba (Cornaredo, Italy), Enamine (Enamine, Latvia) and used, unless otherwise stated, without further purification. Thin-layer chromatography (TLC) was carried out on precoated TLC plates with silica gel 60 F254 (Merk Life Science S.r.l., Milan, Italy). Column chromatography was performed with 1:30 Merck silica gel 60 Å (63–200 μm or 40–63 μm for flash column chromatography) as the stationary phase. Flash chromatographic separations were performed on a Biotage SP1 purification system using flash cartridges prepacked with KP-Sil 32–63 μm, 60 Å silica. Polarimetric measure with polarimeter (POLAX-2L, ATAGO CO., Ltd., Tokyo, Japan).

Melting points were determined with a Büchi apparatus and are uncorrected. ^1^H NMR spectra were recorded on a 500-vnmrs500 Agilent spectrometer (500 MHz) or on a with a Bruker Ascend 500 MHz spectrometer (Bruker Italia S.r.l., Milan, Italy). All chemical shift values are reported in ppm (δ), coupling constants values are reported in Hz (J). Mass spectra were recorded on an HPLC Alliance 2695 (Waters, Milford, MA, USA). High resolution mass spectra (electrospray ionization, ESI-TOF) (HRMS) were recorded on an Agilent 6530 Accurate Mass Q-TOF (mass range 50–3000 *m*/*z*, dry gas nitrogen 10 mL/min, dry heater 325 °C, capillary voltage 4000 V, electrospray ion source in positive or negative ion mode). All spectra were in accordance with the assigned structures. Elemental analyses were determined on a Fisons Instruments Model EA 1108 CHNS-O model analyzer or on a Eurovector Euro EA 3000 analyzer and are within 0.4% of theoretical values. RP-HPLC analysis was performed on an Agilent 1260 Infinity Binary LC System equipped with a diode array detector using a Phenomenex Synergi Fusion-RP column (100 mm × 3 mm, 4 μm particle size). All target compounds were eluted by gradient elution (phase A 0.01% formic acid in water, phase B 0.01% formic acid in ACN; gradient from 10% to 100% B in 10 min) at 0.7 mL/min. Purity of the compounds is >98%.

#### 2-Chloro-5-methyl-*N*-[(1-methyl-4-phenylpiperidin-4-yl)­methyl]­benzamide (**1**)

To a solution of 2-chloro-5-methylbenzoic acid (**8**) (0.06 g, 0.34 mmol) and (1-methyl-4-phenylpiperidin-4-yl)­methanamine (**7**) (0.09 g, 0.44 mmol) in dry DMF (5 mL) PyBOP (0.25 g, 0.48 mmol) and *N*-methylmorpholine (0.12 mL, 0.66 mmol) were added. The reaction mixture was stirred at room temperature overnight. Then, the reaction mixture was diluted with H_2_O (20 mL) and extracted with EtOAc (3 × 20 mL). The organic layers were separated and washed with brine. The collected organic layer was dried over Na_2_SO_4_ and concentrated in vacuo. The crude was purified by column chromatography using CH_2_Cl_2_/MeOH 19:1 (v/v) as eluent to obtain the desired pure compound as a white solid (0.09 g, 75% yield). M.p.: 113–114 °C ^1^H NMR (300 MHz, CDCl_3_) δ 2.02–2.08 (m, 2H), 2.18–2.27 (m, 2H), 2.28 (s, 3H), 2.30 (s, 3H), 2.37–2.48 (m, 2H) 2.65–2.72 (m, 2H), 3.69 (app d, 2H), 5.86 (br t, 1H, D_2_O exchanged), 7.09 (dd; 1H, *J* = 4.9 and 8.9 Hz), 7.17 (d, 1H, *J* = 8.9 Hz), 7.23–7.24 (m, 1H), 7.34 (app s, 3H), 7.39 (app s, 2H). GC-MS *m*/*z* 358 (M^+^+2, 3), 356 (M^+^, 8), 188 (34), 173 (100), 153 (34). Elemental Analysis calcd for C_21_H_25_ClN_2_O: C, 70.67; H, 7.06; N, 7.85. Found: C, 70.59; H, 7.15; N, 7.86.

#### (±)-2-Chloro-3-methoxy-*N*-[2-morpholino-2-[6-(trifluoromethyl)­pyridin-3-yl]­ethylbenzamide (**2**)

The acid **13** (0.06 g, 0.21 mmol,) was dissolved in DMF (2 mL) and reacted with EDC (0.06g, 0.31 mmol), HOBt (0.04 g, 0.31 mmol) and diisopropylethylamine (DIPEA; 110 μL, 0.63 mmol) for 1 h at r. t.. Then, amine **11** (0.06g, 0.21 mmol) was added and allowed to react at r. t. for 12 h. Volatiles were removed under *vacuum,* and the residue was purified on a flash chromatography column by eluting with *n*-hexane n-Hex-EtOAc (90:10 to 70:30). The final compound **2** was obtained as white powder (0.05 g; 52% yield) and as a racemic mixture (at the concentration of 0.044 g/cm^3^, in MeOH, polarimetric analysis gave 0 as rotation angle of polarized light). M.p.: 193–194 °C; ^1^H NMR (500 MHz, CDCl_3_) δ 2.53 (m, 4H, 2xCH_2_), 3.73 (t, *J* = 7.15 Hz, 4H, 2xCH_2_), 3.84 (m, 2H, C*H*_*2*_NH), 3.95 (s, 3H, CH_3_), 3.98 (m, 1H, CH), 6.62 (s, 1H, NH), 7.04 (d, *J* = 6.82 Hz, 1H, H-Ph), 7.19 (d, *J* = 6.91 Hz, 1H, H-Ph), 7.31 (t, *J* = 7.10 Hz, 1H, H-Ph), 7.72 (d, *J* = 7.15 Hz, 1H, H-Ph), 7.86 (d, *J* = 7.19 Hz, 1H, H-Ph), 8.69 (s, 1H, H-Ph). ESI-MS positive ion mode *m*/*z*: [M + H]^+^: 443.9; [M + Na]^+^: 465.8. Elemental Analysis calcd for C_20_H_21_ClF_3_N_3_O_3_: C, 54.12; H, 4.77; F, 12.84; N, 9.47. Found: C, 54.25; H, 4.82; F, 12.70; N, 9.39.

#### *N*-[[2-(Azetidin-1-yl)­pyridin-3-yl]­methyl]-1-(2,3-dichlorophenyl)-1*H*-1,2,4-triazol-5-amine (**3**)

Compounds **20** (0.09 g, 0.30 mmol) and **23** (012 g, 0.73 mmol) were placed in a Parr steel vial and reacted by melting at 140 °C for 12 h. The crude was then purified by column chromatography eluting with c-Hex-DCM (70:30) and 0.1% NH_3_/MeOH. Compound **3** was obtained after crystallization from c-Hex/Et_2_O (0.025 g; 20% yield). M.p.: 82–86 °C; ^1^H NMR (500 MHz, CDCl_3_) δ 2.30 (m, 2H, CH_2_), 4.10 (t, *J* = 7.09 Hz, 4H, 2xCH_2_N), 4.47 (t, *J* = 7.20 Hz, 1H, NH), 4.50 (m, 2H, C*H*_*2*_NH), 6.69 (m, 1H, H-Py), 7.38 (m, 1H, H-Ph), 7.39 (m, 1H, H-Ph), 7.43 (m, 1H, H-Py), 7.65 (m, 1H, H-Py), 7.73 (s, 1H, CH), 8.16 (m, 1H, H-Ph). ESI-MS positive ion mode *m*/*z*: [M + H]^+^: 375.0. Elemental Analysis calcd for C_17_H_16_Cl_2_N_6_: C, 54.41; H, 4.30; N, 22.40. Found: C, 54.49; H, 4.35; N, 22.36.

#### (2,3-Dichlorophenyl)-[3-(4-methoxypyridin-2-yl)-6-methyl-5,6-dihydro-[1,2,4]­triazolo­[4,3-*a*]­pyrazin-7­(8*H*)-yl]­methanone (JNJ54173717)

2,3-Dichlorobenzoyl chloride (**17**) (0.13 g, 0.65 mmol) was added to a stirred solution of amine **16** (0.16 g, 0.65 mmol) and Et_3_N (120 μL, 1.63 mmol) in CH_2_Cl_2_ (20 mL) under nitrogen at 0 °C. The reaction mixture was slowly warmed to room temperature and stirred for 1h. The reaction mixture was quenched with water and extracted with CH_2_Cl_2_ (20 mL). The organic layers were separated dried over Na_2_SO_4_ and concentrated in vacuo. The crude was purified by column chromatography using CH_2_Cl_2_/MeOH 19:1 (v/v) as eluent to obtain the desired pure compound as a white solid (0.13 g, 48% yield). ^1^H NMR (300 MHz, CDCl_3_) δ 1.19 (d, 0.9H, *J* = 7.0), 1.35–1.43 (m, 2H), 3.92 (s, 3H), 4.05–4.13 (m, 0.5H), 4.15–4.24 (m, 0.5H), 4.36–4.48 (m, 0.6H), 4.51–4.63 (m, 0.4H), 4.64–4.78 (m, 0.8H), 4.79–4.80–4.91 (m, 0.2H), 5.01–5.17 (m, 0.6H), 5.47–5.59 (m, 0.4 H), 5.78 (d, 0.3H, *J* = 18.3 Hz), 5.92 (d, 0.2 H, *J* = 18.3 Hz), 6.85 (ddd, 1H, *J* = 12.5, 5.1, 2.6 Hz), 7.14–7.39 (m, 2H), 7.51–7.59 (m, 1H), 7.80–7.89 (m, 1H), 8.30–8.36 (m, 0.4H), 8.42 (d, 0.6H, 5.8 Hz). GC-MS *m*/*z* 419 (M^+^+2, 3), 417 (M^+^, 8), 402 (21), 244 (93), 173 (100), 145 (43), 135 (38). Elemental Analysis calcd for C_19_H_17_Cl_2_N_5_O_2_: C, 54.56; H, 4.10; N, 16.74. Found: C, 54.59; H, 4.05; N, 16.76.

### Biology

#### Cell Culture

HEK293 cells stably transfected with the human P2X7 receptor were grown adherently and supplemented with DMEM/F12 medium containing 10% FBS (fetal bovine serum), 100 U/mL penicillin, 100 μg/mL streptomycin, 0.2 mg/mL Geneticin (G418), and incubated at 37 °C in 5% CO_2_/95% O_2_. Routine Mycoplasma contamination screening was performed using the detection kit supplied by Applied Biological Materials (Richmond, Canada).^49^

#### Transfection of HEK-293 with P2X7R and P2X7-E496A

The plasmids pcDNA3-P2X7R WT and pcDNA3-P2X7-E496A were transformed into *E. coli* DH5α competent cells (Thermo Fisher Scientific) and purified using the GeneJET Plasmid Midiprep Kit (Thermo Fisher Scientific). To develop stable HEK-293 with P2X7R and P2X7-E496A cell lines, HEK-293 cells were plated at a density of 3 × 10^6^ cells in 10 mL growth medium in 100 mm Petri dishes, and incubated at 37 °C overnight. Cells were transfected with 17 μg of pcDNA3-P2X7R WT or pcDNA3-P2X7-E496A using Lipofectamine 3000 (Thermo Fisher Scientific) in Opti-MEM medium without serum. Vector-expressing cells were selected using Geneticin (G418). After transfection, cells were placed in normal DMEM/F12 growth medium. After 1 day, cells were detached with trypsin/EDTA and replated into DMEM/F12 growth medium containing Geneticin (0.8 mg/mL) and cultured for 25 days. Surviving cell clones were picked out and propagated separately in 60 mm Petri dishes in the same medium with 0.8 mg/mL Geneticin (G418). To suppress reversion of the phenotype, all subsequent cell culture was carried out in DMEM/F12 growth medium as described above, supplemented with 0.2 mg/mL Geneticin (G418).^50^

#### Functional Activity Assay at Human P2X7R

The functional activity of the tested compounds was assessed by measuring the changes in intracellular calcium concentration *[Ca* ^*2+*^ *]*_*i*_ in HEK-293 cells stably transfected with human P2X7R. The changes were assessed using the ratiometric fluorescent probe Fura-2/AM (Invitrogen–Thermo Fisher Scientific) and a Cary Eclipse fluorescence spectrophotometer (Agilent Technologies, Milan, Italy), following previously established procedures.^51^ In brief, 2 × 10^6^ cells were incubated with 2 μM Fura-2/AM for 20 min at 37 °C in the presence of 250 μM sulfinpyrazone, using a saline solution composed of 125 mM NaCl, 5 mM KCl, 1 mM MgSO_4_, 1 mM NaH_2_PO_4_, 20 mM HEPES, 5.5 mM glucose, 5 mM NaHCO_3_, and 1 mM CaCl_2_ (pH 7.4). Fluorescence emission was recorded at 505 nm, with excitation wavelengths set at 340 and 380 nm. Dose–response curves were generated and analyzed using GraphPad Prism (GraphPad, La Jolla, California, USA). The data are shown as mean ± standard deviation (SD).

#### Radioligand Binding Assays at Human, Rat and E496A Human Mutant P2X7 Receptor

Unless otherwise stated, all compounds and media for cell culture were purchased from Euroclone S.p.A. (Milan, Italy).

#### Membrane Preparation

Cell membranes were prepared from HEK293 cells stably transfected with human, rat or E496A human mutant P2X7R receptor by mechanically detaching the cells from Petri and resuspending them in a cold hypotonic buffer (5 mM Tris/HCl, 2 mM EDTA, pH 7.4; Sigma-Aldrich, Milan, Italy). The resulting cell suspension was homogenized using an Ultra-Turrax (IKA-WERKE GmbH &Co. KG, Staufen Germany; 2 × 15 s at maximum speed) and the homogenate was centrifuged at 1,000 × g for 10 min at 4 °C. The supernatant was subsequently centrifuged 100,000 × g for 30 min at 4 °C. The resulting pellet, containing the membrane proteins, was resuspended in 50 mM Tris/HCl, pH 7.4. Protein concentration was determined using the Bradford method with the BCA Protein Assay Kit (Thermo Fisher Scientific, Waltham, Massachusetts, USA). The membrane preparations were aliquoted, flash-frozen in liquid nitrogen, and stored at – 80 °C until use.

#### Radioligand Binding

Dissociation constant (K_D_ -value) of the radioligand [^3^H]-JNJ-64413739 (RC-TRITEC AG, Teufen, Switzerland, Lot. N. 2304–28–90) was measured in saturation binding experiments. The affinity (K_i_ values) of tested compounds was determined in radioligand competition experiments.

For saturation binding increasing concentrations of the radioligand (60–0.25 nM) were incubated in a total volume of 250 μL containing 25 μg of membrane proteins in the specific buffer. The nonspecific binding was determined in the presence of JNJ-47965567 100 μM (Cat. N. 5299, Tocris)

In competition experiments, the wells contained 1 nM [^3^H]-JNJ64413739, the tested compounds at different concentrations and 25 μg of membrane proteins in a final volume of 200 μL. The nonspecific binding was determined in the presence of JNJ47965567 100 μM.

In both experiments, samples were incubated for 3 h at room temperature. Following incubation, they were filtered through a 96-well filter plate (UniFilter GF/C, PerkinElmer) using the FilterMate Cell Harvester (PerkinElmer) and subsequently washed three times with cold distilled water. The filter plates were then dried at 40 °C for 30 min. After drying, 20 μL of scintillation liquid (Microscint-20, PerkinElmer) was added to each well. Radioactivity was measured using a MicroBeta^2^ Scintillation Counter (PerkinElmer). Binding data were analyzed by nonlinear regression using GraphPad Prism 8 (GraphPad Software, San Diego, CA, USA).

#### Human and Rat Liver Microsome Stability

Test compounds were preincubated at 37 °C with human or rat liver microsomes (Tebu-Bio, Milan, Italy) (1.0 mg/mL microsomal protein) at a 10 μM final concentration in 100 mM potassium phosphate buffer (pH 7.4) for 10 min. Metabolic reactions were initiated by the addition of the NADPH regenerating system (containing 10 mM NADP, 50 mM glucose-6-phosphate, and 10 unit/mL glucose-6-phosphate dehydrogenase, final glucose-6-phosphate dehydrogenase concentration, 1 unit/mL, Tebu-Bio, Milan, Italy). Aliquots were removed at 0, 5, 15, 30, 60, and 120 min and immediately mixed with an equal volume of cold acetonitrile containing the internal standard. Quenched samples were centrifuged at 4500 rpm for 15 min and the supernatants were injected for quantification analysis. Samples (20 μL) were analyzed by using an Agilent 1100 (Agilent, Santa Clara, United States) equipped with a variable wavelength detector and controlled via OpenLab ChemStation software (Agilent, Santa Clara, United States). Chromatographic separation was achieved using a Synergi Fusion-RP column (100 × 3 mm, 4 μm, 80 Å; Phenomenex, Torrance, United States) at 25 °C. The mobile phase consisted of 0.1% TFA acetonitrile (solvent A) and 0.1% TFA water (solvent B), and was delivered at a flow rate of 0.7 mL/min with the following gradient profile: 0–10 min, linear gradient from 10% to 100% A; 10–11 min, linear gradient from 100% to 10% A; 11–16 min, isocratic at 100% A. Concentrations were quantified by measuring the area under the peak.

The *in vitro* half-life (*t*_1/2_) was calculated using the expression *t*_1/2_ = 0.693/b, where b is the slope found in the linear fit of the natural logarithm of the fraction remaining of the parent compound vs incubation time.^52^ *In vitro* half-life was then used to calculate the intrinsic plasma clearance (CL_int_) according to the following equation:

$${\mathrm{CL}}_{\mathrm{int}}\frac{0.693}{\mathrm{in}\;\mathrm{vitro}\;t_{1/2}}\times \frac{1}{\mathrm{mg}/\mathrm{mL}\;\mathrm{microsomal}\;\mathrm{protein}}$$

#### Permeability Studies

Bidirectional transport studies were performed as previously described,^53,54^ using the human brain microvascular endothelial cell line hCMEC/D3 and Madin–Darby Canine Kidney (MDCK) cells retrovirally transfected with human MDR1 cDNA (MDCKII–MDR1). The flux of fluorescein isothiocyanate–dextran (FD4, Sigma-Aldrich, Italy) and diazepam was used to assess monolayer integrity and barrier function. FD4 fluorescence was measured using a Tecan Infinite M200 plate reader at excitation and emission wavelengths of 485 and 535 nm, respectively.

The amount of compound in each chamber was quantified by RP-HPLC analysis using an Agilent 1100 (Agilent, Santa Clara, United States) equipped with a variable wavelength detector and controlled via OpenLab ChemStation software (Agilent, Santa Clara, United States). Chromatographic separation was achieved using a Synergi Fusion-RP column (100 × 3 mm, 4 μm, 80 Å; Phenomenex, Torrance, United States). The mobile phase consisted of acetonitrile (solvent A) and water (solvent B), and was delivered at a flow rate of 0.7 mL/min with the following gradient profile: 0–10 min, linear gradient from 10% to 100% A; 10–11 min, linear gradient from 100% to 10% A; 11–16 min, isocratic at 100% A. Standard calibration curves were prepared at maximum absorption wavelength of each compound using PBS as solvent and were linear (r^2^ = 0.999) over the range of tested concentration (from 0.5 to 100 μM). Each compound was tested in triplicate.

The apparent permeability coefficient (*P*_*app*_) was calculated according to following equation:

$$P_{app}=\left(\frac{V_{a}}{A\times t}\right)\times \left(\frac{{\left[Drug\right]}_{acceptor}}{{\left[Drug\right]}_{0}}\right)$$

where *V*_*a*_ is the volume of the acceptor well (mL), *A* is the surface area of the membrane (cm^2^), *t* is the total transport time (s), [Drug]_acceptor_ is the drug concentration in the acceptor chamber, and [Drug]_0_ is the initial drug concentration in the apical chamber.

The efflux ratio (ER) was determined using the following equation:

$$ER=\left(\frac{P_{app,BA}}{P_{app,AB}}\right)$$

where *P*_*app,AB*_ and *P*_*app,BA*_ represent the apparent permeability coefficients for apical-to-basolateral and basolateral-to-apical transport, respectively (cm/s).

#### Brain Free Fraction Measurements

Brain free fraction measurements were performed in triplicate at a single concentration of 25 μM, following 4 h of equilibrium dialysis. Commercial male Sprague–Dawley rat brain homogenates (Sekisui Xenotech, Kansas City, United States) were prepared in Tris-HCl/KCl buffer (pH 7.4) at 25% w/v. A volume of 120 μL of brain homogenate (homogenized with 75% Tris-HCl 50 mM, KCl 150 mM, pH 7.4) was spiked with the test compounds at a final concentration of 25 μM (5% DMSO), and incubated for at least 30 min at 37 °C under gentle shaking prior to loading into the retentate chamber of a rapid equilibrium dialysis (RED) device insert (8 kDa MWCO, Thermo Scientific Pierce RED Device, Waltham, United States).

A total of 100 μL of the spiked biological matrix was loaded into the sample chamber (red) of the RED insert, and 350 μL of Tris-HCl buffer (50 mM, KCl 150 mM, pH 7.4) was added to the buffer chamber (white). The units were sealed and incubated at 37 °C under gentle orbital shaking for 4 h using an Inheco Single Plate Incubator Shaker.

At the end of the dialysis, 50 μL aliquots were withdrawn from both chambers and precipitated with 150 μL of cold acetonitrile (3 volumes). Samples were then incubated on ice for 30 min and centrifuged at 10,000 rpm for 10 min at 4 °C. Supernatants were filtered through 4 mm, 0.45 μm syringe filters and analyzed by HPLC (injection volume: 20 μL) using the chromatographic conditions described for permeability studies.

Quantification of compound concentrations was carried out using calibration curves generated from standard solutions of the test compounds dissolved in methanol at the following concentrations: 0.5, 1, 2.5, 5, 10, 25, 50, and 100 μM. Linear regression was applied to derive the calibration equation, which was subsequently used to quantify the concentrations of RED-processed samples.

The unbound fraction (f_u_) of tested compounds in rat brain tissue homogenate (f_u, tissue homogenate_) was calculated by the Eqs. 1) or 2):

$$fu,tissue\;homogenate=\frac{\left[C\;buffer\;chamber\right]}{\left[C\;sample\;chamber\right]}$$ 1

$$fu,tissue\;homogenate=\frac{\left[Area\;buffer\;chamber\right]}{\left[Area\;sample\;chamber\right]}$$ 2

The f_u, tissue homogenate_ determined from diluted tissue homogenates was converted using Eq. 3 to obtain undiluted unbound fraction in the brain tissue (f_u, tissue_). D in Eq. 3 represents the -fold dilution of tissues (Df = 1 + 3 = 4).

$$Undiluted\;fu,tissue=\frac{fu,\mathrm{hom}}{Df-\left(Df-1\right)\left(fu,\mathrm{hom}\right)}$$ 3

#### In Vitro Selectivity Profiling

The receptors, enzymes, and ion channels included in the selectivity profiling were selected among those available in the CNS SafetyScreen panel from Eurofins Discovery (https://emea.eurofinsdiscovery.com/catalog/cns-safetyscreen-panel-fr/P411, last accessed December 21, 2025). The experimental conditions can be found at the same url.

#### Displacement Studies in Human Meningioma Samples

[^3^H]-JNJ64413739 was synthesized and obtained from Tritec AG, Teufen, Switzerland. The specific activity was 81.4 Ci/mmol (3012 GBq/mmol) with radiochemical concentration of 1 mCi/mL at the day of synthesis. The study was approved by the Danish Science Ethics Committee: approval H-19089882 applies, where informed consent was obtained from the patients preoperatively. The study was performed in accordance with the ethical standards as laid down in the 1964 Declaration of Helsinki and its later amendments or comparable ethical standards.

Human meningioma was sectioned at 20 μm and mounted on Superfrost slides. Slides were preincubated twice for 10 min at room temperature in a 50 mM Tris-HCl buffer (pH 7.4) containing 0.5% bovine serum albumin (BSA). Slides were then incubated for 120 min at room temperature on a rotator in the same buffer supplemented with 5 mM MgCl_2_, 2 mM EGTA. For the saturation study, duplicate sections were incubated in concentrations spanning from 0.5 to 100 nM of the radioligand, and nonspecific binding was experimentally determined in the presence of 10 μM JNJ64413739. Specific binding was calculated by subtracting the nonspecific binding from the total binding.

For the displacement study, 30 nM [^3^H]-JNJ64413739 was mixed with either the cold ligand itself or with the tested compounds. The concentrations used for the displacement study ranged from 0.2 nM to 2 μM for tested compounds. Control sections were incubated with 30 nM [^3^H]­JNJ-64413739 in the absence of any test compounds.

The slides were washed twice in an ice-cold preincubation buffer for 10 min and briefly rinsed in an ice-cold distilled water, air-dried, and placed overnight in a paraformaldehyde chamber at 4 °C. After fixation, the glass slides were air-dried and kept in a silica gel desiccator for 60 min to remove any leftover moisture. Glass slides were exposed to phosphor plates for 2 days at 4 °C, then scanned using Amersham Typhoon IP Biomolecular Imager (GE healthcare, Chicago, USA). Resulting autoradiograms were analyzed with ImageJ software (Version 2.9.0, NIH).

Quantitative analysis of receptor binding was performed by measuring the mean of optical density (OD) across the whole meningioma section. Tritium standards were used to convert OD values to radioactivity (nCi/mg) using the Rodbard calibration curve. Values were corrected for radioligand decay and expressed as bound radioligand (fmol/mg tissue equivalent). Relative binding (%) was calculated for each condition by normalizing to the control section, and inhibition curves were generated accordingly.

## Supplementary Material



## Notes

Synthetic procedures of intermediates **6–23**; Western Blot analysis procedure; Saturation curves of the binding studies at human and rat P2X7R and at E496A mutant P2X7R (Figure S1); dose response curve of functional activity of compounds **2** and **3** at hP2X7R (Figure S2); selectivity panel of compound **2** (Table S1) (PDF)

## Abbreviations

CNS: central nervous system
DIPEA: *N*,*N*-diisopropylethylamine
EDC: 1-ethyl-3-(3-dimethylaminopropyl)­carbodiimide
HOBt: hydroxybenzotriazole
NBS: *N*-bromosuccinimide
PET: positron emission tomography
PyBOP: benzotriazol-1-yloxy-tris­(pyrrolidino)­phosphonium hexafluorophosphate
TMSCN: trimethylsilylcyanide
TSPO: 18 kDa translocator protein

## References

[1] Ransohoff R. M. (2016). How neuroinflammation
contributes to neurodegeneration. Science.

[2] Shastri A., Bonifati D. M., Kishore U. (2013). Innate immunity
and neuroinflammation. Mediators Inflamm..

[3] Kwon H. S., Koh S. H. (2020). Neuroinflammation in neurodegenerative
disorders: the
roles of microglia and astrocytes. Transl. Neurodegener..

[4] Jain P., Chaney A. M., Carlson M. L., Jackson I. M., Rao A., James M. L. (2020). Neuroinflammation
PET Imaging: Current Opinion and
Future Directions. J. Nucl. Med..

[5] Chauveau F., Winkeler A., Chalon S., Boutin H., Becker G. (2025). PET imaging
of neuroinflammation: any credible alternatives to TSPO yet?. Mol. Psychiatry.

[6] Ametamey S. M., Honer M., Schubiger P. A. (2008). Molecular
imaging with PET. Chem. Rev..

[7] Willmann J. K., van Bruggen N., Dinkelborg L. M., Gambhir S. S. (2008). Molecular imaging
in drug development. Nat. Rev. Drug Discovery.

[8] Alam M. M., Lee J., Lee S. Y. (2017). Recent
Progress in the Development of TSPO PET Ligands
for Neuroinflammation Imaging in Neurological Diseases. Nucl. Med. Mol. Imaging.

[9] Zhang L., Hu K., Shao T., Hou L., Zhang S., Ye W., Josephson L., Meyer J. H., Zhang M. R., Vasdev N., Wang J., Xu H., Wang L., Liang S. H. (2021). Recent
developments on PET radiotracers for TSPO and their applications in
neuroimaging. Acta Pharm. Sin. B.

[10] Cumbers G. A., Harvey-Latham E. D., Kassiou M., Werry E. L., Danon J. J. (2024). Emerging
TSPO-PET Radiotracers for Imaging Neuroinflammation: A Critical Analysis. Semin. Nucl. Med..

[11] Liu Y. D., Chang Y. H., Xie X. T., Wang X. Y., Ma H. Y., Liu M. C., Zhang H. M. (2025). PET Imaging
Unveils Neuroinflammatory
Mechanisms in Psychiatric Disorders: From Microglial Activation to
Therapeutic Innovation. Mol. Neurobiol..

[12] Lee N., Choi J. Y., Ryu Y. H. (2024). The development
status of PET radiotracers
for evaluating neuroinflammation. Nucl. Med.
Mol. Imaging..

[13] Zhao Y. F., Tang Y., Illes P. (2021). Astrocytic and Oligodendrocytic P2X7
Receptors Determine Neuronal Functions in the CNS. Front. Mol. Neurosci..

[14] Janks L., Sharma C. V. R., Egan T. M. (2018). A central role for
P2X7 receptors
in human microglia. J. Neuroinflammation..

[15] Bhattacharya A., Biber K. (2016). The microglial ATP-gated
ion channel P2X7 as a CNS drug target. Glia.

[16] Morgan J., Alves M., Conte G., Menéndez-Méndez A., de Diego-Garcia L., de Leo G., Beamer E., Smith J., Nicke A., Engel T. (2020). Characterization of the Expression
of the ATP-Gated P2X7 Receptor Following Status Epilepticus and during
Epilepsy Using a P2X7-EGFP Reporter Mouse. Neurosci.
Bull..

[17] Janssen B., Vugts D. J., Funke U., Spaans A., Schuit R. C., Kooijman E., Rongen M., Perk L. R., Lammertsma A. A., Windhorst A. D. (2014). Synthesis and initial preclinical evaluation of the
P2X7 receptor antagonist [^11^C]­A-740003 as a novel tracer
of neuroinflammation. J. Labelled Comp. Radiopharm..

[18] Fantoni E. R., Dal Ben D., Falzoni S., Di Virgilio F., Lovestone S., Gee A. (2017). Design, synthesis and
evaluation
in an LPS rodent model of neuroinflammation of a novel 18F-labelled
PET tracer targeting P2X7. EJNMMI Res..

[19] Green M., Hutchins G., Fletcher J., Territo W., Polson H., Trussell H., Wissmann C., Zheng Q.-H., Gao M., Wang M., Glick-Wilson B. (2018). Distribution
of the P2X7-receptor-
targeted ^11^C-GSK1482160 radiopharmaceutical in normal human
subjects. J. Nucl. Med..

[20] Hagens M. H. J., Golla S. S. V., Janssen B., Vugts D. J., Beaino W., Windhorst A. D., O’Brien-Brown J., Kassiou M., Schuit R. C., Schwarte L. A., de Vries H. E., Killestein J., Barkhof F., van Berckel B. N. M., Lammertsma A. A. (2020). The P2X7
receptor tracer [^11^C]­SMW139 as an in vivo marker of neuroinflammation
in multiple sclerosis: a first-in man study. Eur. J. Nucl. Med. Mol. Imaging..

[21] Rikken R. M., van de Giessen E., Brumberg J., Aarnio R., Joling M., Forsberg
Morén A., Kerstens V., Moein M. M., Nag S., Halldin C., Fazio P., Roos D. S., Berendse H. W., Kassiou M., Wahlroos S., Haaparanta-Solin M., Oikonen V., Schuit R. C., Boellaard R., Windhorst A. D., Jacobs A. H., Lammertsma A. A., Rinne J. O., Varrone A., Golla S. S. V. (2025). Imaging Proinflammatory
Microglia in Parkinson Disease Using [^11^C]­SMW139 PET: A
Multicenter Study. J. Nucl. Med..

[22] Van
Weehaeghe D., Koole M., Schmidt M. E., Deman S., Jacobs A. H., Souche E., Serdons K., Sunaert S., Bormans G., Vandenberghe W., Van Laere K. (2019). [^11^C]­JNJ54173717, a novel P2X7 receptor radioligand as marker for neuroinflammation:
human biodistribution, dosimetry, brain kinetic modelling and quantification
of brain P2X7 receptors in patients with Parkinson’s disease
and healthy volunteers. Eur. J. Nucl. Med. Mol.
Imaging..

[23] Van
Weehaeghe D., Van Schoor E., De Vocht J., Koole M., Attili B., Celen S., Declercq L., Thal D. R., Van Damme P., Bormans G., Van Laere K. (2020). TSPO Versus
P2X7 as a Target for Neuroinflammation: An In Vitro and In Vivo Study. J. Nucl. Med..

[24] Koole M., Schmidt M. E., Hijzen A., Ravenstijn P., Vandermeulen C., Van Weehaeghe D., Serdons K., Celen S., Bormans G., Ceusters M., Zhang W., Van Nueten L., Kolb H., de Hoon J., Van Laere K. (2019). 18F-JNJ-64413739,
a Novel PET Ligand for the P2X7 Ion Channel: Radiation Dosimetry,
Kinetic Modeling, Test-Retest Variability, and Occupancy of the P2X7
Antagonist JNJ-54175446. J. Nucl. Med..

[25] Zhang L., Villalobos A. (2017). Strategies
to facilitate the discovery of novel CNS
PET ligands. EJNMMI Radiopharm. Chem..

[26] Wager T. T., Hou X., Verhoest P. R., Villalobos A. (2016). Central Nervous System Multiparameter
Optimization Desirability: Application in Drug Discovery. ACS Chem. Neurosci..

[27] El
Idrissi I. G., Podlewska S., Abate C., Bojarski A. J., Lacivita E., Leopoldo M. (2024). Structure-Activity Relationships
and Therapeutic Potential of Purinergic P2X7 Receptor Antagonists. Curr. Med. Chem..

[28] Kilburn, J. P. ; Rasmussen, L. K. ; Jessing, M. ; Eldemenky, E. M. ; Chen, B. ; Jiang, Y. ; Hopper, A. Benzamides, WO2014057078A1. 2014

[29] Kilburn, J. P. ; Rasmussen, L. K. ; Jessing, M. ; Eldemenky, E. M. ; Chen, B. ; Jiang, Y. Cyclic amines, US2014/0107335. 2014

[30] Florjancic A. S., Peddi S., Perez-Medrano A., Li B., Namovic M. T., Grayson G., Donnelly-Roberts D. L., Jarvis M. F., Carroll W. A. (2008). Synthesis
and in vitro activity of 1-(2,3-dichlorophenyl)-N-(pyridin-3-ylmethyl)-1H-1,2,4-triazol-5-amine
and 4-(2,3-dichlorophenyl)-N-(pyridin-3-ylmethyl)-4H-1,2,4-triazol-3-amine
P2X7 antagonists. Bioorg. Med. Chem. Lett..

[31] Rudolph D. A., Alcazar J., Ameriks M. K., Anton A. B., Ao H., Bonaventure P., Carruthers N. I., Chrovian C. C., De Angelis M., Lord B., Rech J. C., Wang Q., Bhattacharya A., Andres J. I., Letavic M. A. (2015). Novel methyl substituted 1-(5,6-dihydro-[1,2,4]­triazolo­[4,3-a]­pyrazin-7­(8H)-yl)­methanones
are P2X7 antagonists. Bioorg. Med. Chem. Lett..

[32] Karasawa A., Kawate T. (2016). Structural basis for
subtype-specific inhibition of
the P2X7 receptor. Elife.

[33] Mikkelsen J. D., Aripaka S. S., Kaad S., Pazarlar B. A., Pinborg L., Finsen B., Varrone A., Bang-Andersen B., Bastlund J. F. (2023). Characterization of the Novel P2X7
Receptor Radioligand
[3H]­JNJ-64413739 in Human Brain Tissue. ACS
Chem. Neurosci..

[34] Adinolfi E., Callegari M. G., Ferrari D., Bolognesi C., Minelli M., Wieckowski M. R., Pinton P., Rizzuto R., Di Virgilio F. (2005). Basal activation of the P2X7 ATP receptor elevates
mitochondrial calcium and potential, increases cellular ATP levels,
and promotes serum-independent growth. Mol.
Biol. Cell.

[35] Guo C. R., Sheng D., Li J. Y., Li T. T., Yao J. B., Zhang R., Huang Y., Zhao Y. Y., Wang D. P., Chen J., Li J., Wang J., Zhou Y., Shen C., Jin F., Cao P., Hattori M., Liu H., Yu Y. (2025). Understanding interspecies
drug response variations
between human and rodent P2X7 receptors. Nat.
Commun..

[36] Jiang L. H., Baldwin J. M., Roger S., Baldwin S. A. (2013). Insights into the
Molecular Mechanisms Underlying Mammalian P2X7 Receptor Functions
and Contributions in Diseases, Revealed by Structural Modeling and
Single Nucleotide Polymorphisms. Front. Pharmacol..

[37] Pegoraro A., Grignolo M., Ruo L., Ricci L., Adinolfi E. (2024). P2X7 Variants
in Pathophysiology. Int. J. Mol. Sci..

[38] Andrejew R., Oliveira-Giacomelli Á., Ribeiro D. E., Glaser T., Arnaud-Sampaio V. F., Lameu C., Ulrich H. (2020). The P2X7 Receptor:
Central Hub of Brain Diseases. Front. Mol. Neurosci..

[39] Kolb H. C., Barret O., Bhattacharya A., Chen G., Constantinescu C., Huang C., Letavic M., Tamagnan G., Xia C. A., Zhang W., Szardenings A. K. (2019). Preclinical
Evaluation and Nonhuman
Primate Receptor Occupancy Study of 18F-JNJ-64413739, a PET Radioligand
for P2X7 Receptors. J. Nucl. Med..

[40] Lindberg A., Chassé M., Varlow C., Pees A., Vasdev N. (2023). Strategies
for designing novel positron emission tomography (PET) radiotracers
to cross the blood-brain barrier. J. Labelled
Comp. Radiopharm..

[41] Hitchcock S. A. (2012). Structural
modifications that alter the P-glycoprotein efflux properties of compounds. J. Med. Chem..

[42] Nagar S., Korzekwa K. (2012). Commentary: nonspecific protein binding versus membrane
partitioning: it is not just semantics. Drug
Metab. Dispos..

[43] Di L., Umland J. P., Chang G., Huang Y., Lin Z., Scott D. O., Troutman M. D., Liston T. E. (2011). Species independence
in brain tissue binding using brain homogenates. Drug Metab. Dispos..

[44] Lemoine L., Gillberg P. G., Svedberg M., Stepanov V., Jia Z., Huang J., Nag S., Tian H., Ghetti B., Okamura N., Higuchi M., Halldin C., Nordberg A. (2017). Comparative
binding properties of the tau PET tracers THK5117, THK5351, PBB3,
and T807 in postmortem Alzheimer brains. Alzheimers
Res. Ther..

[45] Urbina-Treviño L., von Mücke-Heim I. A., Deussing J. M. (2022). P2X7 Receptor-Related
Genetic Mouse Models - Tools for Translational Research in Psychiatry. Front. Neural Circuits..

[46] Sluyter R., Adriouch S., Fuller S. J., Nicke A., Sophocleous R. A., Watson D. (2023). Animal Models for the Investigation
of P2X7 Receptors. Int. J. Mol. Sci..

[47] Maurya R., Sharma A., Naqvi S. (2025). Decoding NLRP3
Inflammasome Activation
in Alzheimer’s Disease: A Focus on Receptor Dynamics. Mol. Neurobiol..

[48] Zanoni M., Sarti A. C., Zamagni A., Cortesi M., Pignatta S., Arienti C., Tebaldi M., Sarnelli A., Romeo A., Bartolini D., Tosatto L., Adinolfi E., Tesei A., Di Virgilio F. (2022). Irradiation causes senescence, ATP
release, and P2X7
receptor isoform switch in glioblastoma. Cell
Death Dis..

[49] Szymczak B., Pegoraro A., De Marchi E., Grignolo M., Maciejewski B., Czarnecka J., Adinolfi E., Roszek K. (2025). Retinoic acid-induced
alterations enhance eATP-mediated anti-cancer effects in glioma cells:
Implications for P2X7 receptor variants as key players.. Biochim. Biophys. Acta Mol. Basis Dis..

[50] Abate C., Ferorelli S., Niso M., Lovicario C., Infantino V., Convertini P., Perrone R., Berardi F. (2012). 2-Aminopyridine
derivatives as potential σ(2) receptor antagonists. ChemMedChem..

[51] Adinolfi E., Cirillo M., Woltersdorf R., Falzoni S., Chiozzi P., Pellegatti P., Callegari M. G., Sandonà D., Markwardt F., Schmalzing G., Di Virgilio F. (2010). Trophic activity
of a naturally occurring truncated isoform of the P2X7 receptor. FASEB J..

[52] Chao P., Uss A. S., Cheng K. C. (2010). Use of intrinsic clearance for prediction
of human hepatic clearance. Expert Opin. Drug
Metab. Toxicol..

[53] Sommonte F., Arduino I., Iacobazzi R. M., Laera L., Silvestri T., Lopedota A. A., Castegna A., Denora N. (2024). Microfluidic development
of brain-derived neurotrophic factor loaded solid lipid nanoparticles:
An in vitro evaluation in the post-traumatic brain injury neuroinflammation
model. J. Drug Del. Sci. Technol..

[54] Racaniello G. F., Balenzano G., Arduino I., Iacobazzi R. M., Lopalco A., Lopedota A. A., Sigurdsson H. H., Denora N. (2024). Chitosan and Anionic Solubility Enhancer
Sulfobutylether-β-Cyclodextrin-Based
Nanoparticles as Dexamethasone Ophthalmic Delivery System for Anti-Inflammatory
Therapy. Pharmaceutics.

