# Supplementary material for: A Holistic Approach to Identifying a Positron Emission Tomography (PET) Tracer Candidate for In Vivo Imaging of Purinergic P2X7 Receptor in Neuroinflammation
Source: ACS Pharmacol Transl Sci. 2026 Mar 29;9(4):997–1009. doi: 10.1021/acsptsci.5c00820 (PMC13077496; doi:10.1021/acsptsci.5c00820)
Supplement: Supplementary file 1 [file pt5c00820_si_001.pdf]

SUPPORTING INFORMATION

A holistic approach to identifying a Positron Emission Tomography (PET) tracer candidate for in vivo imaging of purinergic P2X7 receptor in neuroinflammation

Imane Ghafir El Idrissi,[a†] Andrea Spinaci,[b†] Daniele Vitone,[a] Francesca Intranuovo,[a] Mauro Niso,[a] Leonardo Brunetti,[a] Beatrice Francucci,[b] Burcu A. Pazarlar,[c,d] Kristin H. Magnusdottir,[c,d] Eleonora Paradies,[e] Carlo Marya Thomas Marobbio,[f] Ludovica Ricci,[e] Marianna Grignolo,[g] Rosa Maria Iacobazzi,[a] Gabriella Marucci,[b] Diego Dal Ben,[b] Catia Lambertucci,[b] Rosaria Volpini[b], Nunzio Denora,[a] Elena Adinolfi[g], Jens D. Mikkelsen,[c,d] Michela Buccioni,[b] Enza Lacivita,[a]* Marcello Leopoldo[a]

[a]Dipartimento di Farmacia-Scienze del Farmaco, via Orabona, 4 70125 Bari (Italy)
[b]Scuola di Scienze del Farmaco e dei Prodotti della Salute, Università degli Studi di Camerino, via Madonna delle Carceri, s.n.c. 62032 Camerino (Italy)
[c]Neurobiology Research Unit, University Hospital Rigshospitalet, Copenhagen 2100, Denmark
[d]Institute of Neuroscience, University of Copenhagen, Copenhagen 2200, Denmark
[e]CNR Institute of Biomembranes, Bioenergetics and Molecular Biotechnologies (IBIOM), 70125 Bari, Italy
[f]Dipartimento di Bioscienze, Biotecnologie e Ambiente, via Orabona, 4 70125 Bari (Italy)
[g]Dipartimento di Scienze Mediche, Sezione di Medicina Sperimentale, Università degli Studi di Ferrara, via Luigi Borsari, 46, 44121, Ferrara (Italy)

*Corresponding author: Enza Lacivita – Dipartimento di Farmacia – Scienze del Farmaco, Università degli Studi di Bari Aldo Moro, via Orabona, 4, 70125, Bari, Italy.
E-mail: enza.lacivita@uniba.it;

Table of Contents

Synthetic procedures of intermediates **6-23** S3
Western Blot analysis procedure S9
Figure S1. Saturation curves of the binding studies at human and rat P2X7R and at E496A mutant P2X7R. S10
Figure S2. Dose response curve of functional activity of compounds **2** and **3** at hP2X7R S11
Table S1. Selectivity panel of compound **3** S12

Chemistry

General conditions

All reagents, solvents or silica were purchased from Merck (Milan, Italy), Organics BV (Fisher Scientific GmbH, Geel, Belgium), Alfa Aesar (Thermo Fisher, Kandel), Carlo Erba (Cornaredo, Italy), Enamine (Enamine, Latvia) and used, unless otherwise stated, without further purification. Thin-layer chromatography (TLC) was carried out on pre-coated TLC plates with silica gel 60 F254 (Merk Life Science S.r.l., Milan, Italy). Column chromatography was performed with 1:30 Merck silica gel 60 Å (63-200 μm or 40-63 μm for flash column chromatography) as the stationary phase. Flash chromatographic separations were performed on a Biotage SP1 purification system using flash cartridges pre-packed with KP-Sil 32−63 μm, 60 Å silica.

Polarimetric measure with polarimeter (POLAX-2L, ATAGO CO., LTD., Tokyo, Japan). Melting points were determined with a Büchi apparatus and are uncorrected. ^{1}H NMR spectra were recorded on a 500-vnmrs500 Agilent spectrometer (500 MHz) or on a Bruker Ascend 500 MHz spectrometer (Bruker Italia S.r.l., Milan, Italy). All chemical shift values are reported in ppm (δ), coupling constants values are reported in Hz (J). Mass spectra were recorded on an HPLC Alliance 2695 (Waters, Milford, MA, USA). High resolution mass spectra (electrospray ionization, ESI-TOF) (HRMS) were recorded on an Agilent 6530 Accurate Mass Q-TOF (mass range 50-3000 m/z, dry gas nitrogen 10 mL/min, dry heater 325 °C, capillary voltage 4000 V, electrospray ion source in positive or negative ion mode). All spectra were in accordance with the assigned structures. Elemental analyses were determined on a Fisons Instruments Model EA 1108 CHNS-O model analyzer or on a Eurovector Euro EA 3000 analyzer and are within 0.4% of theoretical values. RP-HPLC analysis was performed on an Agilent 1260 Infinity Binary LC System equipped with a diode array detector using a Phenomenex Synergi Fusion-RP column (100 mm x 3 mm, 4 μm particle size). All target compounds were eluted by gradient elution (phase A 0.01% formic acid in water, phase B 0.01% formic acid in ACN; gradient from 10% to 100% B in 10 min) at 0.7 mL/min. Purity of the compounds is > 98%.

1-Methyl-4-phenylpiperidine-4-carbonitrile (6).

A suspension of NaH (60% dispersion in mineral oil, 0.24 g, 10 mmol) in anhydrous DMF (3 mL) was cooled at -10 °C, then a solution of phenylacetonitrile **4** (0.23 g, 2 mmol) in the same solvent was added dropwise. The mixture was stirred at the same temperature for 30 min, then a solution of 2-chloro-N-(2-chloroethyl)-N-methylamine (**5**) (1.1 g, 7.2 mmol) in anhydrous DMF (5 mL) was added dropwise. The reaction mixture was stirred at 70 °C overnight. After cooling, the reaction mixture was quenched with few drops of IPA and then with H_2O (30 mL). The mixture was extracted with AcOEt (3x20 mL). The combined organic layers were washed with brine, dried over Na_2SO_4, and concentrated under reduced pressure. The crude residue was purified by column chromatography using CH_2Cl_2/MeOH 19:1, (v/v) as eluent. The desired compound was obtained as yellowish oil (0.3 g, 75% yield). ^{1}H NMR (300 MHz, $CDCl_3$) δ 2.10-2.21 (m, 4H), 2.43 (s, 3H), 2.48-2.56 (m, 2H), 2.99 (d, 2H, *J*= 13 Hz), 7.32-7.38 (m, 1H), 7.39-7.45 (m, 2H), 7.49-7.54 (m, 2H). GC-MS m/z 201(M^++1, 13), 200 (M^+, 100), 199 (70), 173 (131).

(1-Methyl-4-phenylpiperidin-4-yl)methanamine (7).

To a solution of the nitrile **6** (0.28 g, 1.4 mmol) in ethanolic NH_3 (20 mL, 2 M), Nickel-Raney was added and the mixture was saturated with H_2 at 5 atm pressure. The mixture was stirred at 40 °C for 22 h. Then, the reaction mixture was filtered through a Celite pad and the filtrate was concentrated *in vacuo* to afford the target amine (colorless oil) in quantitative yield. ^{1}H NMR (300 MHz, $CDCl_3$) δ 1.79-1.89 (m, 4H), 2.18-2.28 (m + s, 7H), 2.55-2.65 (m, 2H), 2.77 (m, 2H), 7.21-7.23 (m, 1H), 7.28-7.39 (m, 4H). GC-MS m/z 205 (M^++1, 1), 204 (M^+, 4), 175 (100), 172 (80), 77 (12).

(±)-2-Morpholino-2-(6-(trifluoromethyl)pyridin-3-yl)acetonitrile (10).

Morpholine (308 μL, 3.57 mmol,) and CH_3COONa (0.32 g, 3.90 mmol) were added to a mixture of 2-(trifluoromethyl)pyridin-5-carboxyaldehyde (**9**) (0.57 g, 3.25 mmol) and trimethylsilylcyanide (TMSCN; 813 μL, 6.49 mmol) in CH_3COOH (10 mL). The mixture was stirred at r. t. for 12 h, and

then volatiles were removed under vacuum. Then, a saturated Na_2CO_3 aqueous solution was added, and the resulting mixture was extracted with EtOAc (3 x 50 mL). The organic phases were separated, dehydrated over anhydrous Na_2SO_4, filtered and evaporated. The residue was purified by flash column chromatography by eluting with c-Hex-EtOAc (95:5) to give the desired compound as white powder (0.69 g; 78% yield). ^1H NMR (500 MHz, $CDCl_3$) δ 2.65 (m, 4H), 3.76 (m, 4H), 4.94 (s, 1H), 7.78 (d, 1H, *J* = 7.51 Hz), 8.11 (d, 1H, *J* = 7.51 Hz), 8.95 (s, 1H). ESI-MS positive ion mode m/z: $[M+H]^+$: 271.9. Elemental analysis calcd for $C_{12}H_{12}F_3N_3O$: C, 53.13; H, 4.46; F, 21.01; N, 15.49. Found: C, 53.20; H, 4.50; F, 20.95; N, 15.41.

(±)-2-Morpholino-2-(6-(trifluoromethyl)pyridin-3-yl)ethan-1-amine (11).

20 mL of a 0.05 M solution of nitrile **10** (0.27 g, 1 mmol) in EtOH were injected into a NanoThales Mini Plus H-CUBE at a flow rate of 0.3 mL/min reacting in a 30 mm Ni-Raney cartridge with H_2 at 30 bar pressure at 70 °C. After the 3rd reduction cycle, the resulting solution was evaporated to dryness and the residue was purified by flash column chromatography eluting with DCM-7N MeOH/NH_3 (99:1 to 95:5) to afford amine **11** (61 mg, 22% yield). ^1H NMR (500 MHz, $CDCl_3$) δ 2.44 (m, 4H), 3.10 (m, 2H), 3.45 (t, 1H, *J* = 6.51 Hz), 3.71 (m, 4H), 7.70 (d, 1H, *J* = 7.99 Hz), 7.84 (dd, 1H, *J* = 1.83 and 8.02 Hz), 8.66 (s, 1H). ESI-MS positive ion mode m/z: $[M+H]^+$: 276.1. Elemental analysis calcd for $C_{12}H_{16}F_3N_3O$: C, 52.39; H, 5.86; F, 20.70; N, 15.26. Found: C, 52.45; H, 5.90; F, 20.59; N, 15.22.

2-Chloro-3-methoxybenzoic acid (13).

A solution of 2-chloro-3-methoxybenzaldehyde (**12**) (0.10g, 0.59 mmol), $NaHCO_3$ (0.12 g, 1.48 mmol) and $KMnO_4$ (0.28 g, 1.77 mmol) in H_2O (5 mL) was heated to 90 °C and stirred for 5 h. The mixture was then cooled to r. t. and acidified to pH 4; it was subsequently extracted with dichloromethane (DCM; 3x50 mL). The organic phases were separated, dehydrated by Na_2SO_4 then filtered, and evaporated under vacuum to obtain the desired compound as a white powder (0.09 g,

81% yield). ^1H NMR (500 MHz, $CDCl_3$) δ 3.88 (s, 3H), 7.27 (m, 2H), 7.38 (m, 1H), 13.37 (brs, 1H). ESI-MS negative ion mode m/z: $[M-H]^-$: 185.1. Elemental analysis calcd for $C_8H_7ClO_3$: C, 51.51; H, 3.78. Found: C, 51.60; H, 3.80.

(*S*)-*t*-Butyl 3-(4-methoxypyridin-2-yl)-6-methyl-5,6-dihydro-[1,2,4]triazolo[4,3-*a*]pyrazine-7(8*H*)-carboxylate (15).

To a solution of (*S*)-*t*-butyl 2-methyl-5-oxopiperazine-1-carboxylate (**14**, 0.2 g, 0.9 mmol) in anhydrous DCM (3 mL) was added trimethyloxonium tetrafluoroborate (0,15 g, 1 mmol) dissolved in the same solvent (3 mL). The reaction mixture was stirred at room temperature for 6 h, then a solution of 4-methoxypyridin-2-ylhydrazide (0.2 g, 1.2 mmol) in anhydrous DCM (5 mL) was added dropwise. The reaction mixture was stirred at room temperature overnight. The solvent was concentrated under reduced pressure and the residue was dissolved in dioxane (5 mL) and saturated $NaHCO_3$ (5 mL). The reaction mixture was heated at 90 °C for 6 h, then the dioxane was removed under reduced pressure and the aqueous layer was extracted with EtOAc (3x10 mL). The combined organic layers were dried with Na_2SO_4, filtered and concentrated under reduced pressure. The crude was purified by column chromatography using CH_2Cl_2/MeOH, 19:1 (v/v) as eluent. The desired compound was obtained as a pale yellow solid (0.28 g, 90% yield). ^1H NMR (300 MHz, $CDCl_3$) δ 1.45 (d, 3H, *J*= 6.4 Hz), 1.49 (s, 9H), 3.92 (s, 3H), 4.28 (dd, 1H, *J*= 4.7 and 14 Hz), 4.50 (d, 1H, *J*= 17 Hz), 4.83 (br s, 1H), 4.99-4.92 (m, 1H), 5.22 (d, 1H, *J*= 17 Hz), 6.84 (dd; 1H, *J*= 2.6 and 6.0 Hz), 7.84 (d, 1H, *J*= 2.6 Hz), 8.31 (d, 1H, *J*= 6.2 Hz). HRMS (ESI^+) calcd for $[(C_{17}H_{23}N_5O_3)+Na]^+$: 363.1693, found 363.1695. ESI^+/MS/MS $[M+Na]^+$ *m/z* 263 (100).

(*S*)-3-(4-Methoxypyridin-2-yl)-6-methyl-5,6,7,8-tetrahydro-[1,2,4]triazolo[4,3-*a*]pyrazine (16).

Trifluoroacetic acid (2 mL) was added to a solution of compound (*S*)-**15** (0.270 g, 0.8 mmol) in DCM (10 mL). The reaction mixture was stirred at room temeperature for 45 min, then it was basified with saturated $NaHCO_3$ and extracted with CH_2Cl_2 (2x10 mL). The combined organic

layers were dried with Na_2SO_4, filtered and concentrated under reduced pressure. The crude was purified by column chromatography using CH_2Cl_2/MeOH, 9:1 (v/v) as eluent. The desired compound was obtained as a white solid (0.18 g, 92% yield).). ^{1}H NMR (300 MHz, $CDCl_3$) δ 1.30 (d, 3H, *J*= 6.4 Hz), 2.01 (br s, 1H, D_2O exchanged), 3.12-3.22 (m, 1H), 3.79-3.88 (m, 1H), 3.94 (s, 3H), 4.12 (d, 1H, *J*= 16.5 Hz), 4.32 (d, 1H, *J*= 16.5 Hz), 4.76 (dd, 1H, *J*= 4.0 and 13.4 Hz), 7.02 (dd; 1H, *J*= 2.7 and 6.1 Hz), 7.67 (d, 1H, *J*= 2.7 Hz), 8.49 (d, 1H, *J*= 6.1 Hz). GC-MS m/z 358 (M^++2, 3), 356 (M^+, 8), 188 (34), 173 (100), 153 (34). HRMS (ESI^+) calcd for $[(C_{12}H_{15}N_5O)+Na]^+$: 268.1169, found 268.1163. ESI^+/MS/MS $[M+Na]^+$ *m/z* 268 (100).

1-(2,3-Dichlorophenyl)-1*H*-1,2,4-triazole (19).

A mixture of hydrazine **18** (1.0 g, 5.6 mmol) and formamide (535 mL, 13.44 mmol) was heated in a sealed steel vial up to 150 °C for 16 h. Subsequently the reaction mixture was extracted with H_2O/EtOAc, organic phases were collected, washed with brine, anhydrified over Na_2SO_4 then filtered, and evaporated. The crude was purified by flash column chromatography eluting with n-Hex-EtOAc (98:2 to 80:20) thus obtaining compound **19** (0.94 g; 79% yield). ^{1}H-NMR (500 MHz, $CDCl_3$) δ 7.59 (t, 1H, *J* = 7.12 Hz), 7.65 (d, 1H, *J* = 7.15 Hz), 7.88 (d, 1H, *J* = 7.10 Hz), 8.30 (s, 1H), 9.02 (s, 1H). ESI-MS positive ion mode m/z: $[M+H]^+$: 214.0. Elemental analysis calcd for $C_8H_5Cl_2N_3$: C, 45.10; H, 2.36; N, 19.73. Found: C, 45.20; H, 2.40; N, 19.65.

5-Bromo-1-(2,3-dichlorophenyl)-1*H*-1,2,4-triazole (20).

To a solution of triazole **19** (0.7 g, 3.3 mmol) in CCl_4 (15 mL), NBS (1.76 g, 9.9 mmol) and benzoyl peroxide (40 mg, 0.165 mmol) were added, and the mixture was refluxed for 24 h. Then, the volatiles were removed under vacuum, and the residue was purified by flash column chromatography eluting with c-Hex-EtOAc (98:2 to 94:6) to obtain the desired compound as a white powder (0.58 g; 60% yield). ^{1}H-NMR (500 MHz, $CDCl_3$) δ 7.38 (m, 2H), 7.72 (m, 1H), 8.10 (s, 1H). ESI-MS positive:

[M+H]$^+$: 293.7. Elemental analysis calcd for $C_8H_4BrCl_2N_3$: C, 32.77; H, 1.37; N, 14.35. Found: C, 32.85; H, 1.40; N, 14.39.

2-(Azetidin-1-yl)nicotinonitrile (22).

To a solution of 2-fluoronicotinonitrile (**21**, 1.6 g, 13.10 mmol) in dry THF (15 mL), Et_3N (65.5 mmol, 9 mL) and azetidine hydrochloride (1.1 g, 59.81 mmol) were added at 0 °C, slowly warmed to r. t. and allowed to react for 16 h. The volatiles were removed under vacuum; H_2O was added to the residue, and the mixture was extracted with DCM (3 x 50 mL). The organic phases were collected, dried over anhydrous Na_2SO_4 then filtered and evaporated under vacuum. The crude residue was purified by crystallization using EtOAc and c-Hex to give the desired compound as a yellowish oil (2.0 g, 96% yield). ^{1}H-NMR (500 MHz, $CDCl_3$) δ 2.43 (m, 2H), 4.37 (t, 4H, J = 7.15 Hz), 6.59 (m, 1H), 7.65 (m, 1H), 8.28 (m, 1H). ESI-MS positive ion mode m/z: [M+H]$^+$: 160.2. Elemental analysis calcd for $C_9H_9N_3$: C, 67.92; H, 5.70; N, 26.38. Found: C, 68.00; H, 5.75; N, 26.27.

(2-(Azetidin-1-yl)pyridin-3-yl)methanamine (23).

Compound **22** (0.2 g, 1.25 mmol) was dissolved in 7N methanolic ammonia (NH_3 in MeOH, 5 mL) and placed under hydrogen atmosphere (4 atm) using freshly prepared Ni/Raney, at 50 °C for 6 h. After filtration through celite the residue was purified by flash column chromatography eluting with DCM/NH_3 in MeOH 7N sol. (98:2 to 90:10) to afford amine **23** as a light-yellow solid (0.18 g; 87% yield). ^{1}H-NMR (500 MHz, $CDCl_3$) δ 2.36 (m, 2H), 3.76 (br s, 2H), 4.15 (t, 4H, J = 7.10 Hz), 6.70 (m, 1H), 7.44 (m, 1H), 8.13 (m, 1H). ESI-MS positive ion mode m/z: [M+H]$^+$: 164.2. Elemental analysis calcd for $C_9H_{13}N_3$: C, 66.26; H, 8.02; N, 25.72. Found: C, 66.35; H, 8.05; N, 25.65.

Western Blot analysis on P2X7R-WT and P2X7-E496A transfected HEK-293.

Cells were washed three times with PBS and lysed in buffer containing 0.15 M NaCl, 5 mM EDTA, 1% NP-40, and 10 mM Tris-Cl (pH 7.4). Protein concentration was determined using the Bradford Protein Assay (Bio-Rad). Equal amounts of protein (50 µg) were separated on 10% Criterion TGX Stain-Free™ Gels (Bio-Rad) and electrophoresed at 180 V for 45 min. Gels were activated for 1 min using a ChemiDoc™ XRS+ Imaging System and transferred to midi-size (13.5 × 8 cm) PVDF membranes using the Trans-Blot® Turbo™ Transfer System (Bio-Rad).

Following transfer, membranes were UV-activated (302 nm) to obtain total protein images, which were quantified using the lane volume function in Image Lab™ v6.0 (Bio-Rad). Membranes were blocked and incubated for 1 h at room temperature with primary anti-P2X7 antibody (Sigma, 1:1000) and β-actin as housekeeping (Abcam 1:2000) followed by three washes with TBS and incubation with HRP-conjugated anti-rabbit secondary antibody (Pierce, 1:10,000) for 1 h at room temperature. Immunoreactive bands were visualized using Immobilon™ Western Chemiluminescent HRP Substrate (Millipore).

Densitometric analysis was performed using Image Lab™ v6.0, with normalization to total protein signal or β-actin, as recommended by the manufacturer (Bio-Rad).

Figure S1. Saturation curves of [^{3}H]-JNJ 64413739 at P2X7Rs: A) human P2X7R; B) rat P2X7R; C) E496A human mutant P2X7R, radioligand used up to 100 nM; D) E496A human mutant P2X7R, radioligand used up to 400 nM.*

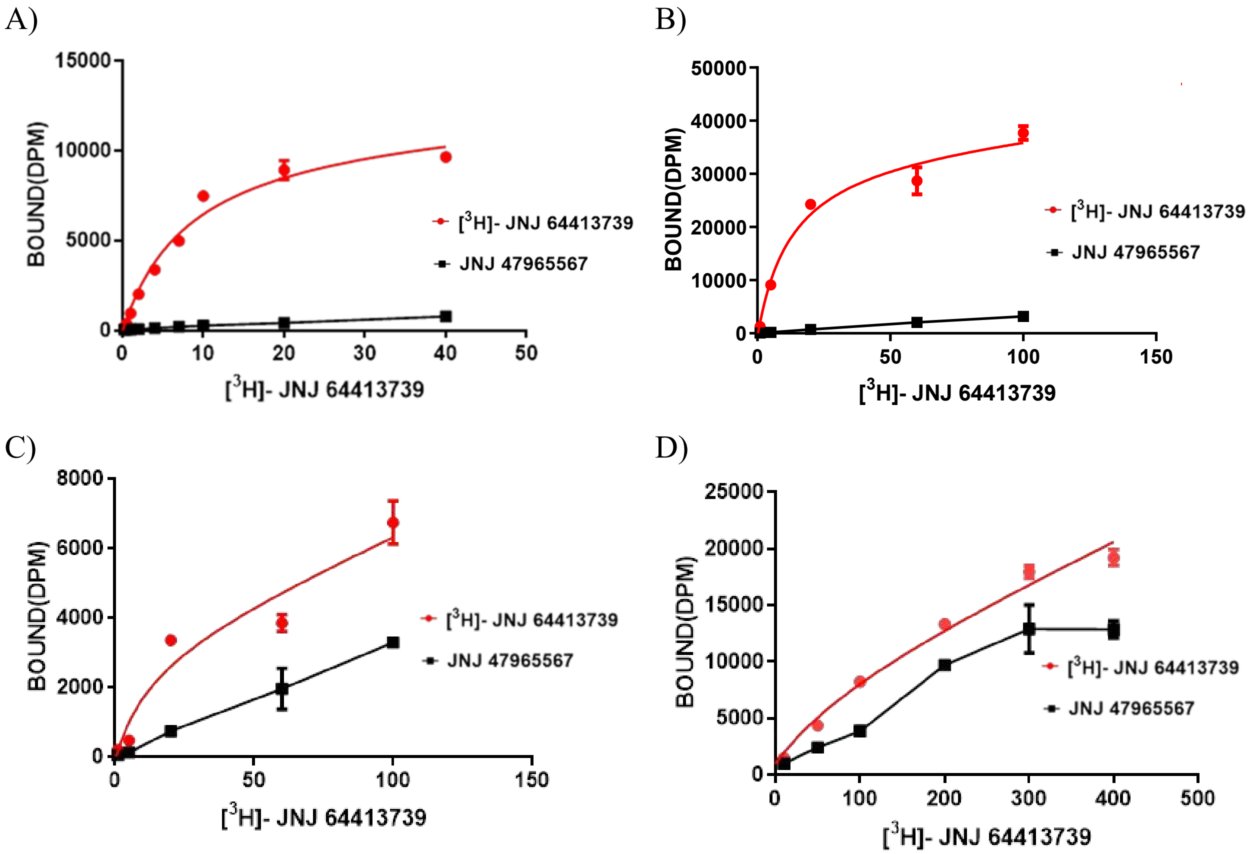

*Concentration nM; DPM = disintegrations per minute.

Curves show saturation at different radioligand concentrations for wild-type and mutant receptors. While [^{3}H]-JNJ64413739 saturated hP2X7R at 40 nM and rP2X7Rs at 100 nM, it did not saturate E496A hP2X7Rs at up to 400 nM. Thus, [^{3}H]-JNJ64413739 cannot be used for radioligand binding assays in E496A hP2X7R.

Figure S2. Dose-response curve of compounds **2** (A) and **3** (B).

A)

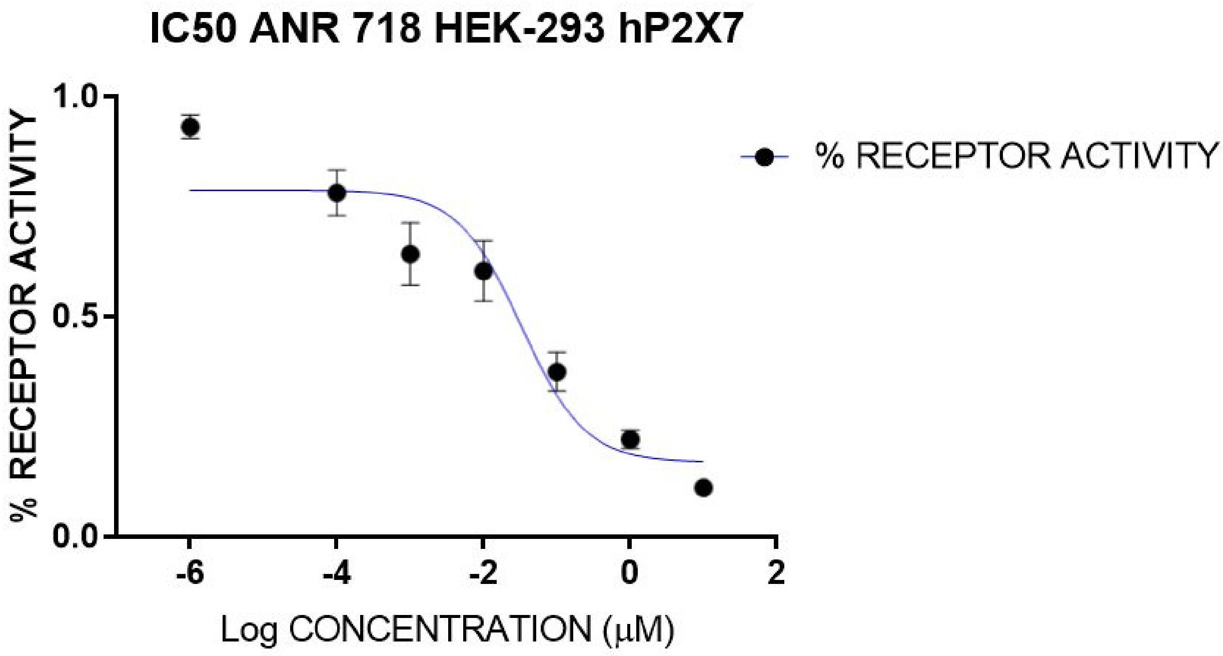

B)

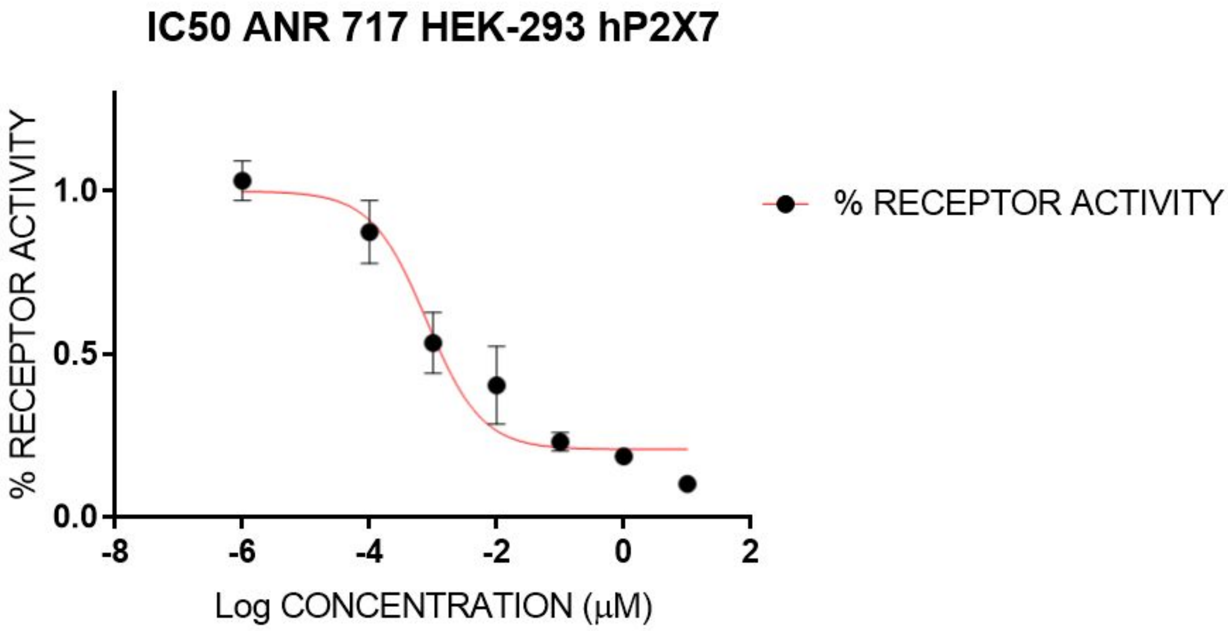

Table S1. Selectivity panel of compound **3** towards CNS receptors and enzymes.

**Receptor/enzyme**	**Species**	**% inhibition @ 10 µM**	**Receptor/enzyme**	**Species**	**% inhibition @ 10 µM**
AchE	human	-6	glycine, strychnine sensitive	rat	3
MAO-A	human	-1	NeuropeptideY1	human	-2
MAO-B	human	1	NeuropeptideY2	human	-6
PDE4D2	human	-1	Nicotinic $\alpha_4\beta_2$, cytisine	human	-5
Androgen	human	11	Nicotinic α_7, bungarotoxin	human	5
Bradykinin B_2	human	-1	Orexin OX_1	human	-2
TSPO	human	9	Orexin OX_2	human	-8
CGRP1	human	-3	Oxytocin	human	2
Ca^{2+} channel L-type	rat	-8	5-HT_3	human	2
CB1	human	6	σ_1	Human	2
CB2	human	-5	Nav1.5	human	-11
CCK2	human	4	Tachykinin NK_1	human	-6
ET_A	human	7	DAT	human	0
ERα	human	-9	GABA-T	rat	-3
$GABA_A$, flunitrazepam	rat	-2	NET	human	3
$GABA_{B1B}$	human	16	SERT	human	-8
Glucorticoid	human	-16	Vasopressin V_{1A}	human	-11
AMPA	rat	5	Adenosin A1	human	5
kainate	rat	3	Adenosin A2A	human	5
$mGlu_5$	human	-16	Adenosin A2B	human	6
NMDA	rat	-7	Adenosin A3	human	8
